# Solution Structure of a Lanthanide‐binding DNA Aptamer Determined Using High Quality pseudocontact shift restraints

**DOI:** 10.1002/chem.202202114

**Published:** 2022-10-01

**Authors:** Witold Andrałojć, Julia Wieruszewska, Karol Pasternak, Zofia Gdaniec

**Affiliations:** ^1^ Institute of Bioorganic Chemistry Polish Academy of Sciences Noskowskiego 12/14 61-704 Poznan Poland

**Keywords:** aptamer, lanthanide ions, NMR spectroscopy, nucleic acids, paramagnetic effects

## Abstract

In this contribution we report the high‐resolution NMR structure of a recently identified lanthanide‐binding aptamer (LnA). We demonstrate that the rigid lanthanide binding by LnA allows for the measurement of anisotropic paramagnetic NMR restraints which to date remain largely inaccessible for nucleic acids. One type of such restraints ‐ pseudocontact shifts (PCS) induced by four different paramagnetic lanthanides ‐ was extensively used throughout the current structure determination study and the measured PCS turned out to be exceptionally well reproduced by the final aptamer structure. This finding opens the perspective for a broader application of paramagnetic effects in NMR studies of nucleic acids through the transplantation of the binding site found in LnA into other DNA/RNA systems.

## Graphical abstract

Long‐range paramagnetic NMR restraints remain largely inaccessible for nucleic acid systems. In this contribution we demonstrate that such restraints can be generated by a lanthanide ion bound to an intrinsic site provided by a DNA aptamer. By solving the 3D structure of the aptamer‐Ln^3+^ complex we provide the structural basis for the introduction of similar sites into other DNA systems.

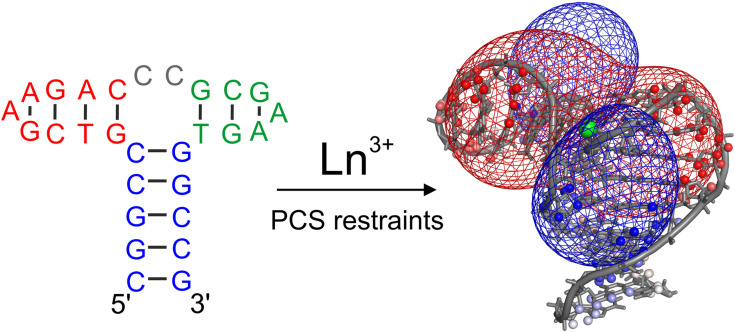

## Introduction

NMR spectroscopy is among the prime experimental techniques providing high‐resolution information about structure and dynamics of proteins and nucleic acids. Between these two biopolymer types, however, NMR studies of nucleic acids are intrinsically more challenging due to factors such as lower proton density, smaller degree of chemical diversity (and thus more spectral overlap) or more difficult access to isotopic enrichment.[^1^, ^2^] On top of that, methodological development efforts in biomolecular NMR are often focused on protein systems, only with time finding their way into nucleic acid studies. One important group of NMR techniques that had already demonstrated their tremendous potential in the field of protein NMR, yet remain largely inaccessible for nucleic acids are the so‐called paramagnetic NMR methods. The presence of a paramagnetic metal ion within a biomolecule has a profound effect on its NMR properties. When such an ion is tightly and rigidly attached to a macromolecule, these spectral changes can be translated into very long‐range NMR structural restraints (up to ∼35 Å), as has been practiced extensively in the field of protein NMR for over 20 years now.[^3^, ^4^, ^5^, ^6^] The long‐range paramagnetic NMR restraints most commonly used in the biomolecular context include: paramagnetic relaxation enhancements (PRE), pseudo‐contact shifts (PCS) and self‐alignment residual dipolar couplings (pRDC). The observation of the latter two effects requires the paramagnetic center to display an anisotropic magnetic susceptibility tensor (**Δχ**≠0), and thus is exclusive to metal ions possessing this property – most notably tripositive lanthanides (the PRE effect can be made accessible using organic radicals instead of metal ions).[^3^, ^4^, ^5^, ^6^] So far, paramagnetism‐based restraints were shown not only to yield better defined structures, but were also demonstrated to greatly facilitate endeavors such as: studies of protein‐protein interactions^[7]^ including identification of minor states,[^8^, ^9^] studies of large‐scale motional processes, like inter‐domain mobility in proteins,[^10^, ^11^] ultrafast assignment of multidimensional NMR spectra^[12]^ or quick establishment of protein‐drug interaction geometries.^[13]^ As most proteins do not contain natural lanthanide binding sites the use of paramagnetic restraints in proteins is only possible thanks to major efforts undertaken to develop methods of lanthanide tagging of proteins.^[5]^ The tagging methods introduced over the years can be divided into two main categories, 1) covalent attachment of lanthanide ion complexes into protein targets[^14^, ^15^] and 2) the introduction of an intrinsic lanthanide binding site directly into protein primary sequence using a specifically designed high‐affinity lanthanide binding peptide (LBP).[^16^, ^17^, ^18^, ^19^] Similarly reliable lanthanide tagging methods for nucleic acids remain yet to be developed. The feasibility of developing a nucleic acid equivalent of the first of the above‐mentioned tagging avenues was recently explored by several groups.[^20^, ^21^, ^22^] On the other hand, attempts at paramagnetic tagging of nucleic acids by exploiting intrinsic lanthanide binding sites have not yet been reported. Such situation may partially stem from the lack of conviction that suitable sites could even be formed by DNA/RNA molecules, given their very different metal ion binding capabilities as compared to proteins. Namely, the strongest metal binding sites in naturally occurring nucleic acid systems are usually characterized by K_d_ values in the 10^−6^–10^−4^ M range[^23^, ^24^] and even artificially obtained metal‐binding aptamers rarely exceed this limit.^[25]^ Affinities in this range would not be enough to achieve quantitative metal ion binding at NMR concentrations (10^−4^–10^−3^ M), making such sites unsuitable for paramagnetic tagging for NMR studies. On top of that, metal ion binding to many sites in nucleic acids occurs without dehydration of the former,^[26]^ leaving the ion in a highly symmetric environment **(Δχ** ≈0) and thus incapable of inducing anisotropic paramagnetic effects – PCS and pRDC.

In the current work we investigate whether a lanthanide‐binding DNA aptamer (abbreviated LnA from now on) identified by Edogun et al.^[27]^ could provide a rare example of a lanthanide binding site with the desired qualities, that could be then transplanted into other nucleic acid systems as a paramagnetic lanthanide tag. The original report^[27]^ employed in vitro selection followed by further construct optimization to produce a 44 nt long DNA molecule capable of binding a single lanthanide ion with a K_d_ of ∼330 nM. Herein we demonstrate that the original aptamer can be significantly shortened without affecting lanthanide affinity and report a high‐resolution NMR structure of a 28 nt long variant, LnA_28. More importantly we demonstrate that a lanthanide ion bound to this system induces large PCS, readily translatable into long‐range structural restraints. Indeed, PCS restraints from four paramagnetic lanthanides were used in the structure determination process itself, yielding an excellent fit to the final structures (Q_factors_∼0.1 for each set) and leading to a significantly better‐defined bundle of structures. Thus, the binding site present in this aptamer is a good candidate for the design of a lanthanide‐binding oligonucleotide tag. Based on the structure we discuss the avenues of further truncation of the prospective tag and approaches to how it could be incorporated into other DNA/RNA systems.

Concurrently, metal ions constitute an important type of targets for aptamer selection efforts and metal sensing by nucleic acid molecular devices (both aptamers and metal‐dependent catalytic DNA/RNA) is a rapidly growing field of research.[^25^, ^28^, ^29^] However, all such metal‐binding oligonucleotide systems are currently discovered by in vitro selection from random libraries, their rational design being hampered by the lack of structural understanding of metal‐nucleic acid interactions in such systems. The solution structure of LnA_28 constitutes the first reported high‐resolution structure of a metal‐binding DNA aptamer and, together with the recently reported structures of 8–17^[30]^ and 10–23^[31]^ DNAzymes, constitutes a starting point for deeper mechanistic understanding of metal‐sensing oligonucleotide devices.

## Results and Discussion

**Figure 1:**
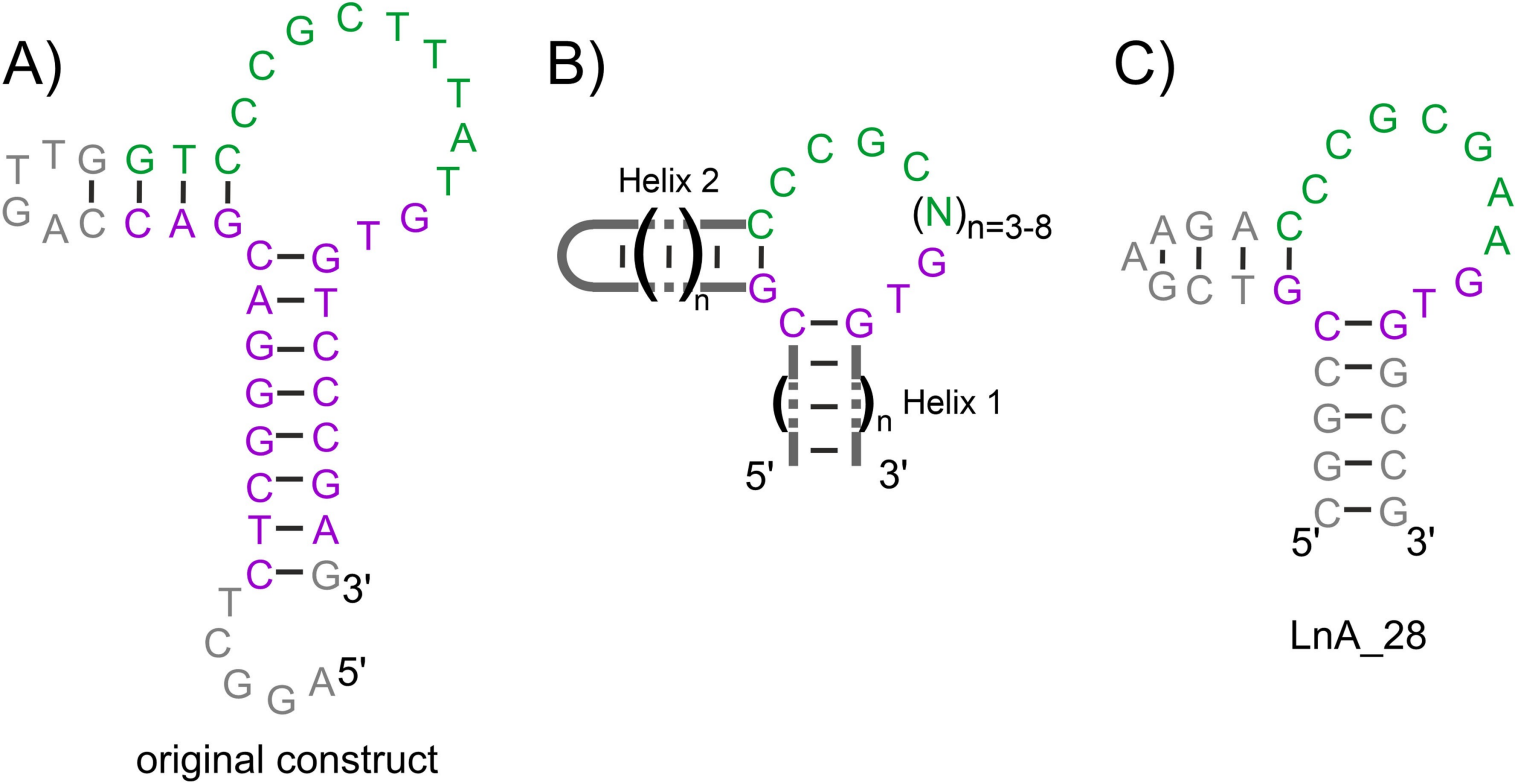
The lanthanide binding aptamer. A) The ‘Ln_aptamer’ – original LnA construct,^[27]^ B) The tentative consensus structure, C) The shortened LnA_28 construct. The residues are color‐coded according to their role in the original SELEX experiment: constant region – magenta, variable region – green, introduced only during post‐SELEX optimization – gray.

**Figure 2:**
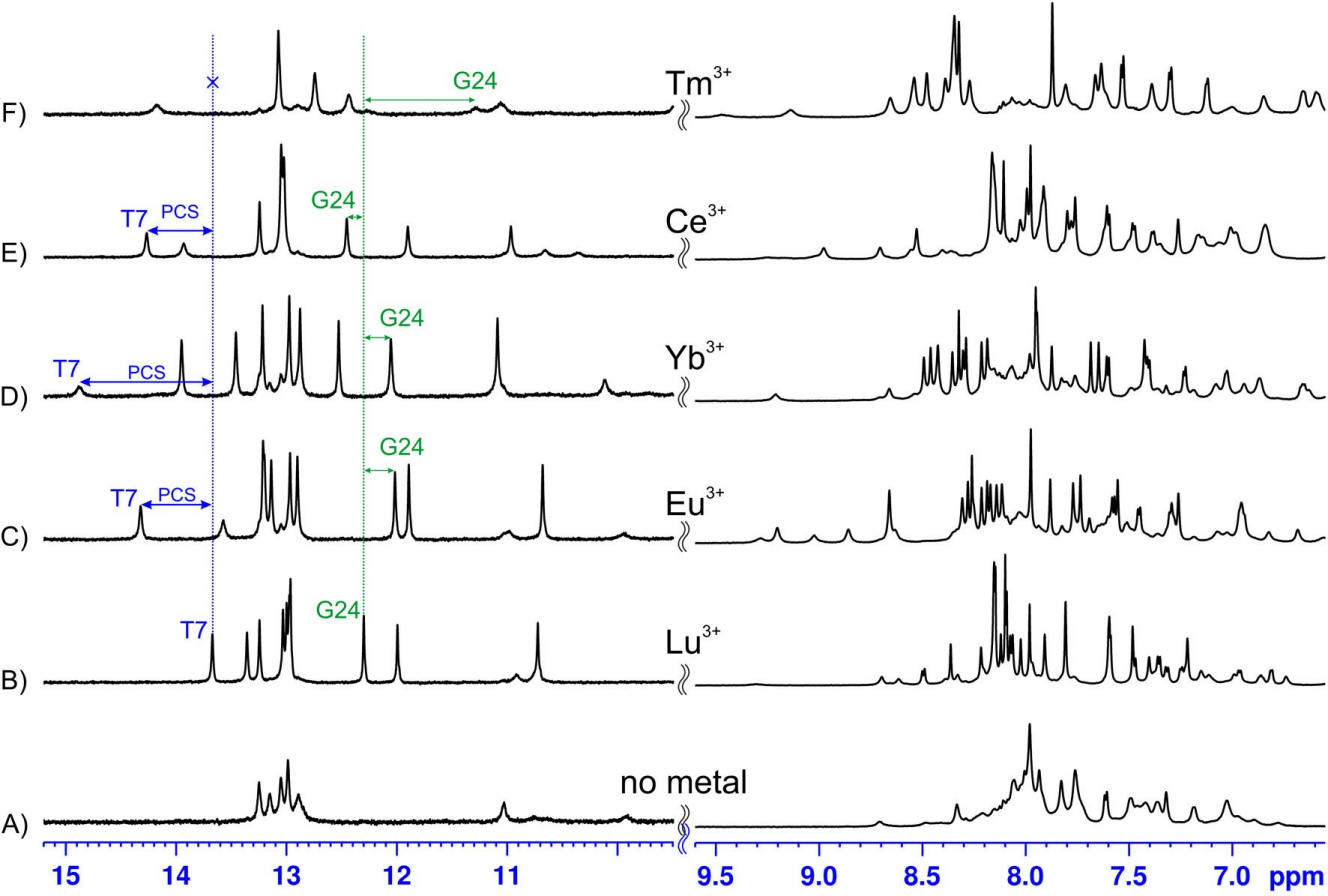
Imino and aromatic regions of 1D NMR spectra of LnA_28 either free (A) or in presence of: Lu^3+^ (B), Eu^3+^ (C), Yb^3+^ (D), Ce^3+^ (E), or Tm^3+^(F).

**Aptamer construct optimization and validation**. As it is typical for SELEX experiments, the original study^[27]^ that isolated LnA has yielded not a single DNA construct capable of binding lanthanide ions, but instead a family of 22 such molecules. The sequence similarities between these lanthanide binding DNAs provide preliminary insights into which sequential and structural elements are likely crucial for metal binding and which ones can be safely removed or substituted to obtain an optimized construct for the NMR study. Figures 1a and 1b present the secondary structure prediction for a 44 nt long variant of LnA, characterized in the original study^[27]^ and the consensus sequence that can be proposed for all the LnA constructs reported in that study, respectively. With these sequential and structural guidelines in mind, we have performed a stepwise sequential optimization towards a shortened construct of LnA without affecting the lanthanide‐binding capability. This last feature was monitored throughout the optimization using 1D NMR complemented by CD spectroscopy, as these techniques turned out to be greatly sensitive to metal binding to LnA (see Supporting Information and Figure 2a–b). The intermediate steps of the optimization process are covered in the Supporting Information and only the final, 28 nt long, construct is presented here (LnA_28; Figure 1c). It features significant changes to Helices I and II, aimed at preserving strong hybridization propensity despite their reduced lengths. The tetra loop originally capping Helix II was substituted with a GAA triloop ‐ the most stable loop sequence known in DNA, thanks to the formation of a sheared G−A pair.[^32^, ^33^] As for the interhelical loop region, the sequential variant from construct 21 in^[27]^ was selected due to its shortest length.

The shortened LnA_28 construct retains unaltered lanthanide affinity, as demonstrated by fitting the CD spectral response observed upon Eu^3+^ titration using a 1 : 1 interaction model (Figure S1a–d). Both the original LnA and LnA_28 yield apparent K_d_ values in the low‐hundred nanomolar range (∼100‐300 nM depending on the wavelength selected for fitting; e. g. Figure 2c–d), confirming high‐affinity binding reported in.^[27]^ CD titrations with two other lanthanide ions (Ce^3+^ and Lu^3+^) were also performed (Figure S2), yielding very similar apparent K_d_ values, confirming the previous observation^[27]^ that the aptamer is capable of interacting with the entire lanthanide ion series. The metal‐free and metal‐saturated forms of the two constructs were also subjected to UV melting analysis (Figure S1e–f). In both cases the shape of the melting profile turned out to be greatly affected by metal binding, changing from a multistep, non‐cooperative melting over a broad range of temperatures to a single well‐defined melting transition. Such a behavior suggests that upon metal binding the molecule converts from a state in which several secondary structure elements are present, yet do not interact with each other, to one containing a well‐defined tertiary structure, melting in a single transition. Interestingly, when the melting temperatures (T_m_) of the bound aptamers are compared it turns out that, despite having significantly shortened Helices I and II, the LnA_28 construct is actually more thermally stable (T_m_∼65 °C vs. 60 °C for the original LnA).

**Paramagnetic lanthanide ions bound to LnA_28  induce large PCS**. The affinity of LnA_28 towards lanthanide ions is high enough that at oligonucleotide concentrations typical for NMR studies the ion binding should be practically quantitative (at 700 μM oligo:700 μM metal, as used in this work, one expects around 98 % bound and 2 % free species, assuming K_d_=∼300 nM). Figures 2a and 2b show the imino and aromatic regions of the ^1^H NMR spectrum of LnA_28 before and after the addition of 1 molar equivalent of a diamagnetic lanthanide ion, Lu^3+^, respectively. While LnA_28 is clearly partially pre‐structured before the addition of the metal ion (imino protons in Watson‐Crick pairs visible in Figure 2a), the presence of Lu^3+^ induces a significant reshuffling of the imino resonances as well as the appearance of new ones, indicating further structuring. When a paramagnetic lanthanide ion is bound instead, one expects a similar structuring effect and, on top of it, the appearance of additional contributions to the chemical shifts (PCS) and linewidths (PRE) of the oligonucleotide protons. As the Figures 2c–f demonstrate this is indeed the case, with each metal giving rise to a very distinct pattern of PCS, as should be expected based on their very different **Δχ** tensors. Interestingly, when the samples were subsequently transferred to D_2_O, for non‐exchangeable proton assignment the chemical shifts in all the ‘paramagnetic’ samples were subject to clearly noticeable changes, while those of the Lu^3+^ bound sample remained largely unaltered. This observation signifies that for each studied paramagnetic lanthanide its **Δχ** tensor changes when passing from H_2_O to D_2_O. Such a behavior might suggest that the lanthanide ions remain partially hydrated when bound to LnA (and the change in **Δχ** is due to ligand exchange from H_2_O to D_2_O). The PCS measured in D_2_O samples were used in all subsequent analysis.

The resonance assignment of the Lu^3+^ containing sample was achieved using standard methods for non‐isotopically labeled nucleic acids, based mostly on ^1^H‐^1^H through‐space connectivities (NOE), as described in Materials and Methods and exemplified in Figure S3a. The samples containing paramagnetic ions required their own separate resonance assignments (see Figure S3b for Eu^3+^), as the PCS effect significantly reshaped the spectral picture for each consecutive sample (Figure S4). Moreover, each time a significant fraction of the expected peaks was missing, due to shortened coherence lifetimes (PRE). All assigned chemical shifts are gathered in Table S2.

**Figure 3:**
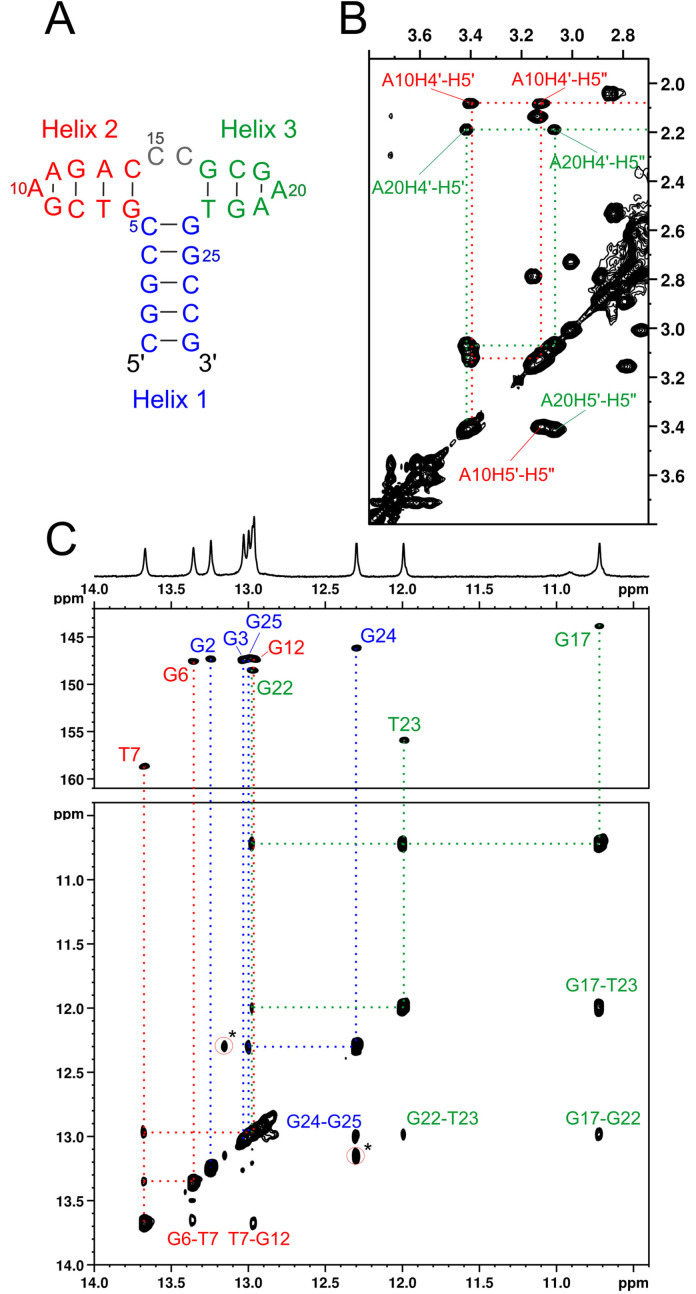
A third helix is present in LnA_28 structure. A) The updated secondary structure, B) H4’ and H5’/H5’’ resonances characteristic to GAA triloops C) The imino region of LnA_28‐ Lu^3+^ complex. Marked with * is an exchange peak with residual metal‐free aptamer.

**The secondary structure of LnA_28 – the presence of a third helix**. While the imino proton patterns observed for the Lu^3+^‐bound LnA_28 confirm the formation of the expected Helices I and II, three additional exchange‐protected imino protons are also observed. Two of them, resonating at 10.72 and 11.99 ppm, are connected by a very strong NOE contact (Figure 3c). Imino chemical shifts in this range, accompanied by a very short inter‐imino distance, are characteristic to a G−T wobble base pairing. The third unexpected imino proton resonates at 12.97 ppm and its NOE contacts, especially the strong NOEs to C18‐NH_2_ group, indicate that it belongs to a residue forming a G : C base pair with C18. This proton also gives rise to imino‐imino contacts to the two other unexpected resonances mentioned above (Figure 3c).

Moreover, the central residue of the G19‐A20‐A21 element displays a set of very unusual proton chemical shifts, namely the A20‐H4’ proton resonating at 2.19 ppm and the A20‐H5’/H5’’ protons at 3.07/3.42 ppm (Figure 3b). Such a chemical shift pattern is very characteristic to GNA triloops, forming a sheared G−A base pair.^[33]^ One such GNA loop was introduced to the LnA_28 construct on purpose to cap Helix II (residues G9‐A10‐A11) and produces a very similar set of characteristic chemical shifts, with A10‐H4’ at 2.09 ppm and the A10‐H5’/H5’’ protons at 3.12/3.40 ppm (Figure 3b). The fact that the G19‐A20‐A21 also gives rise to the chemical shift pattern typical to a GNA loop signifies that not only one but two GAA triloops form in LnA_28 (G9‐A10‐A11 capping Helix II and G19‐A20‐A21 in the supposed ‘loop’ region).

With T23 being the only thymidine available for wobble base pairing these observations can only be explained by the formation of an additional stable helical element, encompassing the residues G17 to T23 and composed of the G17‐T23 wobble base pair, the C18‐G22 Watson‐Crick pair and the G19‐A20‐A21 ultrastable triloop (named Helix III from now on; Figure 3a). Thus, the only actually unpaired residues in the entire LnA_28 structure are C15 and C16, located between Helices II and III.

The third helical element is likely a general feature of LnA and not just the construct studied here, as the residues forming the two observed base pairs are universally conserved in the sequences reported in.^[27]^ Indeed, during preliminary NMR experiments on the original LnA construct we have also observed the strong imino‐imino NOE contact, indicative of a GT pair formation (data not shown). However, both imino resonances involved were significantly broadened by exchange, indicating a lower stability of this element, as expected given the non‐complementary TTTAT sequence capping the helix (Figure 1a). Decreased stability of Helix 3 in the original LnA could also explain its lower T_m_ reported above.

**The high‐resolution structure of LnA_28**. As described in detail in Materials and methods and in the Supporting Information the structure of LnA_28 was calculated in a two‐step procedure, involving initial folding with diamagnetic restraints (NOE and dihedral angles), followed by refinement using both diamagnetic and paramagnetic (PCS) NMR data. All the calculations were performed in the SANDER program from the AMBER18 molecular dynamics suite.^[34]^ The SANDER program already contained a routine to handle the PCS data in structure calculations,^[35]^ yet it was missing the ability to use PCS data originating from several samples simultaneously. Thus, the routine was modified in‐house to provide this functionality, as described in detail in the Supporting Information. After the refinement with all restraints, 15 best structures were selected to form the final NMR bundle, based on their fidelity to both experimental data and ideal covalent geometries. The final bundle of structures is very well defined, with a pairwise RMSD of 0.70 Å, and contains no violations of the NOE (>0.5 Å), dihedral or hydrogen bonding restraints (Table S3). Remarkably, the PCS data are also exceptionally well reproduced, with Q_factors_ of 0.074, 0.068, 0.135 and 0.067 for Eu^3+^, Yb^3+^, Ce^3+^ and Tm^3+^ datasets, respectively (Table S3). The details of PCS fitting and how the usage of these data impacted the final structural bundle are discussed in a separate paragraph below.

**Figure 4:**
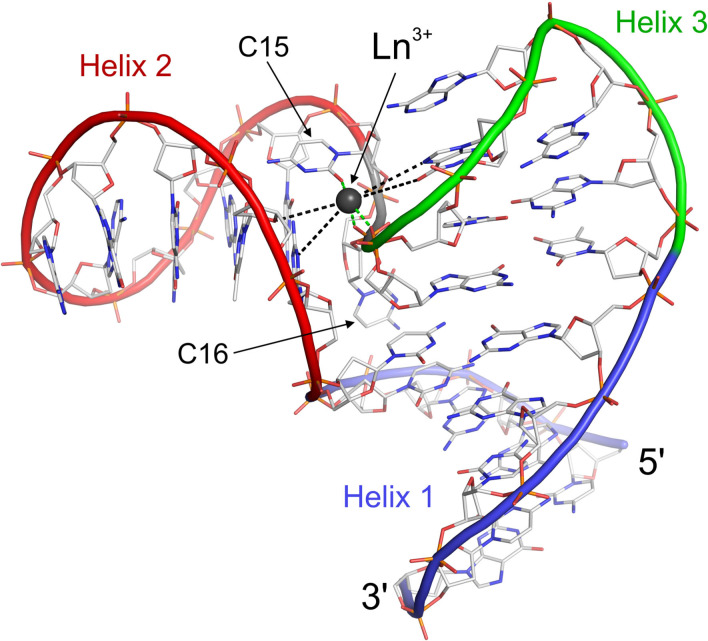
The NMR structure of LnA_28 in complex with a lanthanide ion.

**Figure 5:**
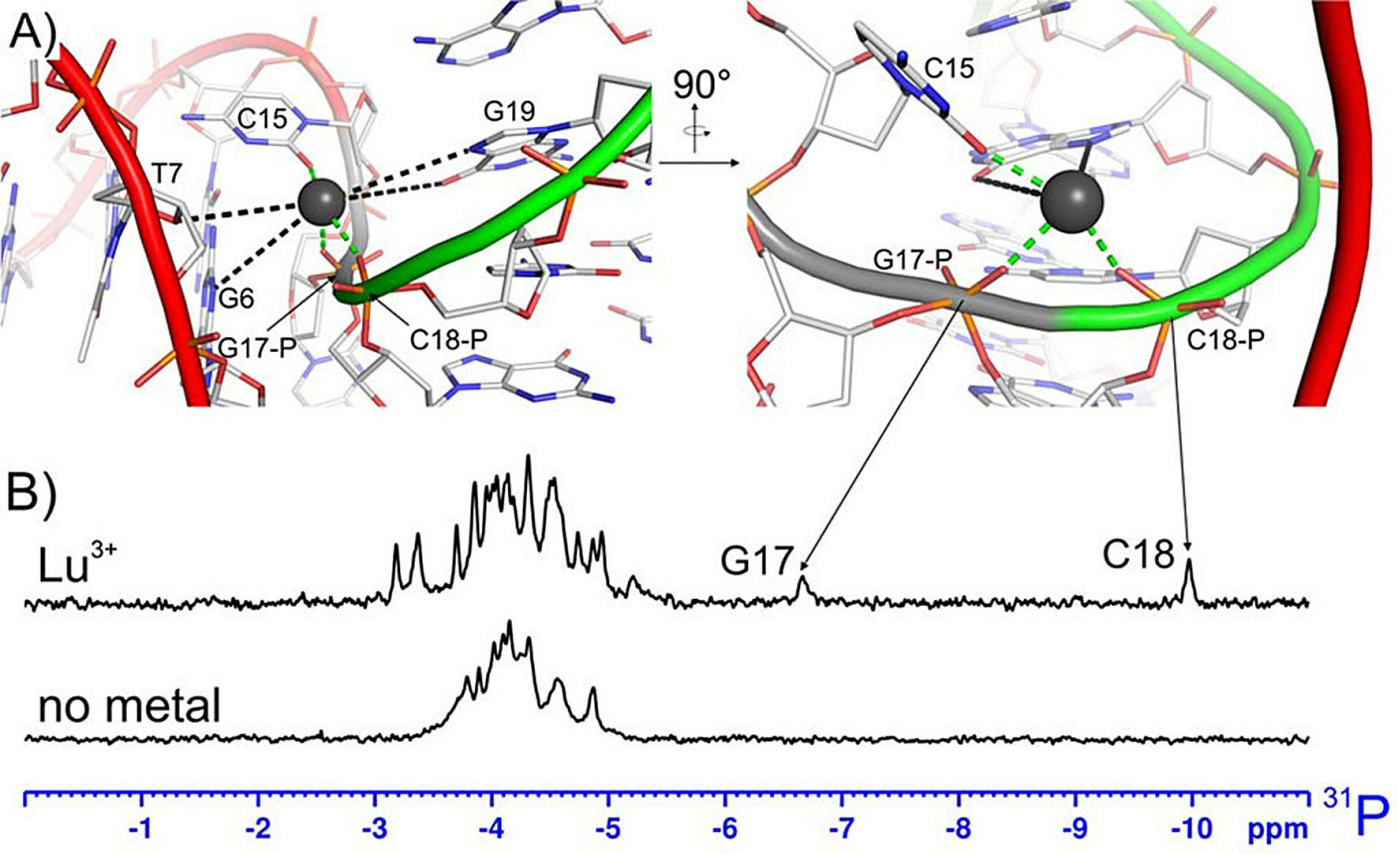
(A) The lanthanide binding site of LnA_28. Direct DNA‐metal interactions marked in green, additional electronegative DNA groups situated <5 Å from the metal in black. (B) The ^31^P NMR spectra of LnA_28 in complex with Lu^3+^ (upper) and free (lower).

A representative structure from the NMR bundle for LnA_28 is presented in Figure 4. The structure features the three helices predicted beforehand in an arrangement in which Helices I and III are coaxially stacked (blue and green in Figure 4), while Helix II (red) propagates at around a right angle from the other two. The two unpaired cytidine residues C15 and C16 interact with the minor grooves of Helices II (C15) and I (C16), most likely further stabilizing the rigid arrangement of the three helices. The peculiar interhelical geometry assumed by LnA_28 leads the C16‐G17‐C18 fragment of the sugar‐phosphate backbone to become buried between the first base pair of Helix II and the Hoogsteen edge of bases forming Helix III (Figures 4 and 5a). Especially, the phosphate group of G17 is located very close to the helical axis of Helix II. Such an arrangement creates a high local density of both negatively charged and electron‐pair donating groups. Interestingly, the PCS data place the lanthanide ion binding site in close proximity of exactly this fragment of the sugar‐phosphate backbone, jammed between the two helices.

**The metal binding site geometry**. Thanks to the PCS data, reporting directly on the position of each nucleus with respect to the metal center, the position of the bound lanthanide ion is very well‐determined within the LnA_28 structural bundle (RMSD=0.11 Å). The obtained structure shows that the lanthanide ion interacts directly (distance <2.5 Å) with three atoms of the DNA molecule: the *pro‐*S oxygen of G17 phosphate group, the *pro‐*R oxygen of C18 phosphate group and the O2 oxygen of the C15 nucleobase (Figure 5a). Thus, lanthanide ion binding to LnA_28 occurs with partial dehydration. The direct interaction between the metal ion and G17 and C18 phosphate groups is probably responsible for the extremely unusual ^31^P chemical shifts observed for these two groups: −6.66 and −9.98 ppm, respectively (Figure 5b), in the diamagnetic Lu^3+^ sample. Four additional electronegative atoms are also located at around 4.5‐5.0 Å from the metal and thus likely belong to its second coordination sphere (Figure 5a). These are: the O6 and N7 atoms from the Hoogsteen edge of G19, the N3 atom of G6 nucleobase and the O4’ atom of the T7 sugar moiety.

**Figure 6:**
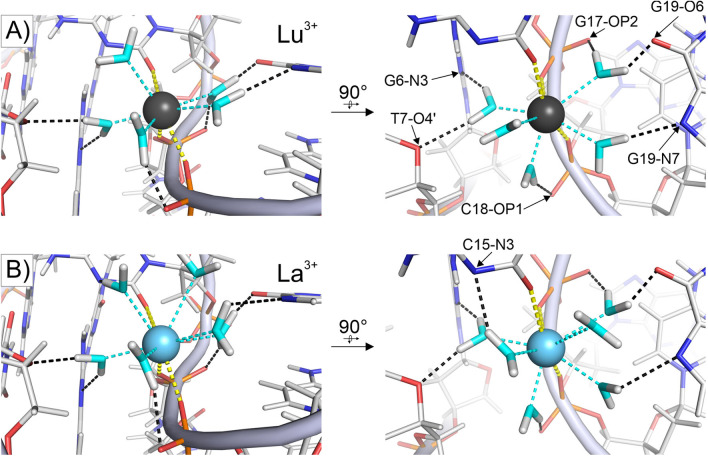
First hydration spheres of Ln^3+^ ions – (A) Lu^3+^ and (B) La^3+^ – bound to LnA_28, explored through MD simulations. Direct DNA‐metal interactions in yellow, H_2_O‐metal interactions in cyan, H_2_O‐DNA hydrogen bonds in black.

As the typical coordination number of lanthanide ions is either 8 or 9, and DNA ligands only fill up 3 of these positions, the rest of the first coordination sphere of a Ln^3+^ ion bound to LnA_28 has to be occupied by water molecules. In order to gain insight into the role of H_2_O molecules in mediating the DNA‐metal interactions, we have studied the behavior of water molecules coordinating lanthanide ions in complex with LnA_28 through the means of molecular dynamics (MD) simulations in explicit solvent. The analysis of the localization of solvent molecules throughout the simulations (see Experimental Section) has demonstrated that ordered hydration waters can be found almost exclusively in the direct vicinity of the bound metal ions (Figure S5). For the LnA_28‐Lu^3+^ complex, this analysis revealed five water molecules directly interacting with the lanthanide ion (coordination number of 8), retaining well‐defined positions throughout the simulation. The MD simulation also supports the idea that the four electronegative atoms at around 4.5–5.0 Å from the metal are indeed involved in the interaction. Namely, these H_2_O molecules form specific, high occupancy H‐bonds with all four atoms (Figure 6a, Table S4). Moreover, the metal‐bound water molecules also form high occupancy hydrogen bonds with oxygen atoms of G17 and C18 phosphate groups that do not directly bind the Lu^3+^ ion (Figure 6a). The MD simulation featuring a La^3+^ ion reports the presence of six water molecules directly coordinating the ion (coordination number of 9), instead of just five as was observed for Lu^3+^ (Figure 6b). This is not surprising as Lu^3+^ is the lanthanide ion with the smallest ionic radius, while La^3+^ has the highest radius in the series. However, despite the different coordination numbers almost the same pattern of water‐DNA hydrogen bonds can be observed (Figure 6a vs. b, Table S4). Additional simulations with Tm^3+^, Eu^3+^ and Ce^3+^ ions have yielded the exact same hydrogen bonding pattern (Table S4). Interestingly, while Ce^3+^ accepted six water molecules in its first coordination sphere, both Tm^3+^ and Eu^3+^ ions demonstrated a less defined coordination number, alternating between states coordinating five and six H_2_O molecules, reflecting their intermediate ionic radii (Table S4).

Overall, the performed simulations suggest the presence of an intricate pattern of interactions between the first hydration sphere of the lanthanide ion and electronegative groups within the DNA molecule, largely independent of the particular lanthanide ion. In total, nine DNA electronegative atoms appear to be involved in lanthanide binding, three through direct coordination and six through hydrogen bonding to metal‐bound water molecules. A level of caution is however necessary when building upon these results, as the assumptions underlying classical MD simulations necessarily lead to inaccuracies in modelling interactions involving highly charged metal ions.[^36^, ^37^]

**Ln^3+^‐Ln_A complex in the light of the current state of knowledge regarding nucleic acid‐lanthanide interactions**. High‐resolution structural information about lanthanide‐nucleic acid complexes is very scarce. A query using the MINAS database^[38]^ reveals only 6 PDB entries containing both a nucleic acid chain and a lanthanide ion or ions.[^39^, ^40^, ^41^, ^42^, ^43^, ^44^] The analysis of these 6 structures suggests that binding without any dehydration of the ion is the dominant type of interaction. For most of these structures, however, we do not have any information regarding the binding affinity of the Ln^3+^ ion to any specific site and some of the sites can actually only be due to specific intermolecular contacts in the crystal lattice and not be populated at all in solution state.^[39]^ Lower resolution techniques, prevalently lanthanide luminescence lifetime measurements, can also be used to estimate the hydration states of Ln^3+^ ions interacting with nucleic acids. Interestingly, such studies paint a somewhat different picture and suggest interaction modes involving two or more inner‐sphere contacts for a wide plethora of sites.[^45^, ^46^, ^47^, ^48^, ^49^, ^50^, ^51^] None of these binding sites, however, were studied using high resolution methods and thus the geometry of the interaction and the identities of the inner sphere ligands remain unknown.

Experiments studying Ln^3+^ ion complexation by free nucleobases have demonstrated that among the four DNA bases only cytosine shown appreciable interactions with Ln^3+^, with the O2 and N3 atoms proposed as the interacting groups.^[52]^ Thus, perhaps the presence of cytidine O2 atom as a direct ligand in LnA‐Ln^3+^ complex is a consequence of this preference observed at single nucleobases level and possibly even a source of the specificity of LnA to lanthanide ions, over a wide range of other di‐ and trivalent metal ions, reported in the original publication.^[27]^

**Figure 7:**
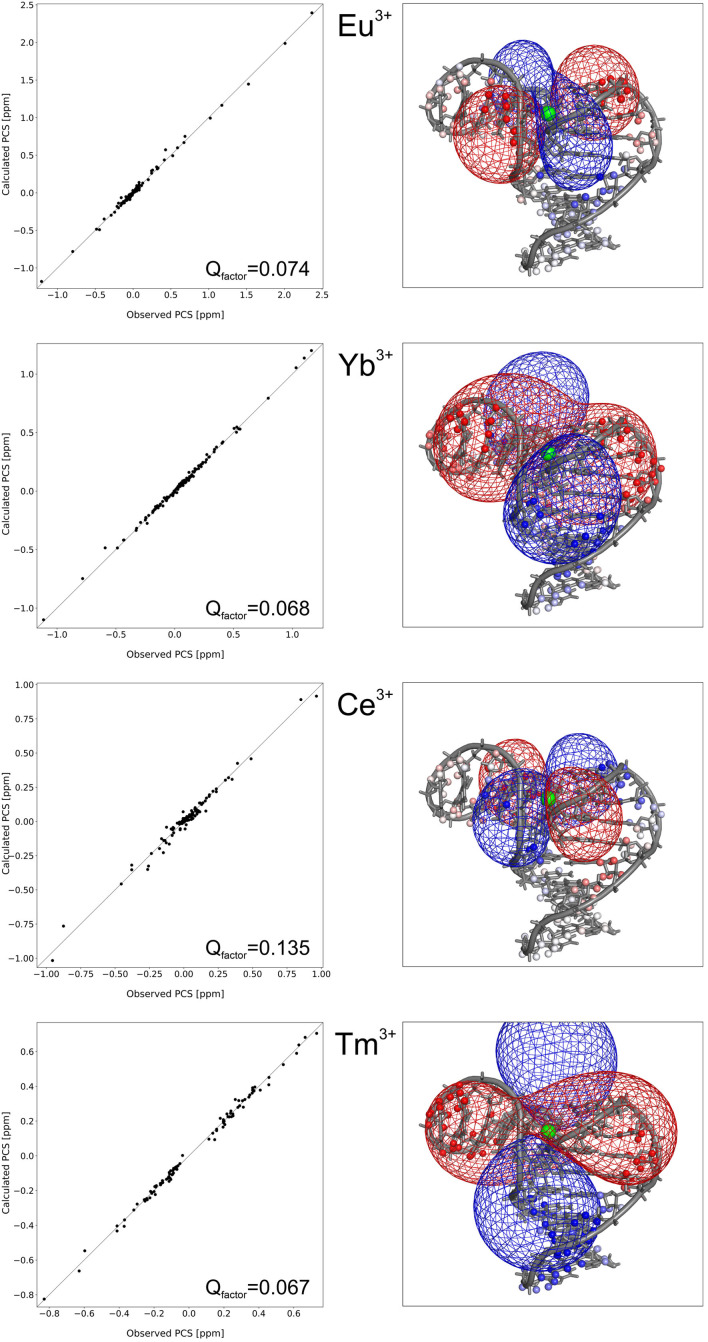
The fitting of PCS data. In the right panels, PCS isosurfaces at +0.2 ppm (red) and −0.2 ppm (blue) are shown as coloured meshed, while the atoms for which PCS were measured experimentally are marked with spheres following the same colour code (fading to white for the smallest PCS values).

**Paramagnetic PCS restraints and their effect on the solved structure**. The final structure reproduces the measured PCS data remarkably well, with Q_factors_ of 0.074, 0.068, 0.135 and 0.067 for Eu^3+^, Yb^3+^, Ce^3+^ and Tm^3+^ datasets, respectively. The fitting of the PCS data is plotted in Figure 7 and the optimized **Δχ** tensor parameters are gathered in Table 1. To better appreciate the agreement between the experimental and back‐calculated data the **Δχ** tensors and the measured PCS are simultaneously visualized on the solved structure in Figure 7. The positive and negative ‘lobes’ of the **Δχ** tensors correspond perfectly to the regions in space in which PCS of that specific sign were experimentally measured. The decreasing magnitude of PCS as the proton‐metal distance increases can also be appreciated. Such a precise reproduction of the PCS measured all throughout the molecule by a **Δχ** tensor signifies that the tertiary structure of LnA_28 is indeed rigid, with the three helices adopting well‐defined positions with respect to each other. If interhelical dynamics were present, the PCS measured within the regions of the molecule subjected to these motions would be averaged over the different conformations and the entire PCS dataset for a given metal would not be fittable to a single **Δχ** tensor. On the other hand, some local dynamics within the first coordination sphere of the lanthanide ion are very likely, given the reduced magnitudes of the **Δχ** tensors (see below). Further cross‐validation of the obtained structure can be provided by PCS measured for ^31^P nuclei of the phosphate groups, that were not used during the structure calculations. As shown in Figure S6 if these data are fitted to the LnA_28 structure (using **Δχ** tensor parameters derived beforehand for ^1^H PCS) Q_factors_<0.2 are obtained for all metals excluding Ce^3+^. This is a very good reproduction taking into account much greater experimental uncertainty of ^31^P shifts derived from the indirect dimension of a ^1^H−^31^P correlation and the fact that these shifts were mostly small (mostly measured for atoms further away from the metal).

**Table 1:** The final Δχ tensor parameters for paramagnetic lanthanide ions bound to LnA_28 and a comparison to their counterparts for protein‐bound ions.

System	**Δχ** tensor paramater	Ce^3+^	Eu^3+^	Tm^3+^	Yb^3+^
LnA_28	Δχ_ax_ (10^−32^ m^3^)	0.96±0.02	1.42±0.01	−3.88±0.03	−2.77±0.02
	Δχ_rh_ (10^−32^ m^3^)	−0.61±0.05	−0.55±0.04	1.62±0.04	1.29±0.02
calbindin D_9k_ ^[53]^	Δχ_ax_ (10^−32^ m^3^)	2.08±0.13	−2.34±0.16	−21.9±0.6	−8.26±0.7
	Δχ_rh_ (10^−32^ m^3^)	0.71±0.21	−1.63±0.28	−20.1±1.0	−5.84±0.6
Ln‐DOTA−M7FPy^[54]^	Δχ_ax_ (10^−32^ m^3^)	3.3±0.04	4.1±0.05	83±0.9	23±0.3
	Δχ_rh_ (10^−32^ m^3^)	1.7±0.03	2.4±0.05	8±0.8	8±0.2

**Figure 8:**
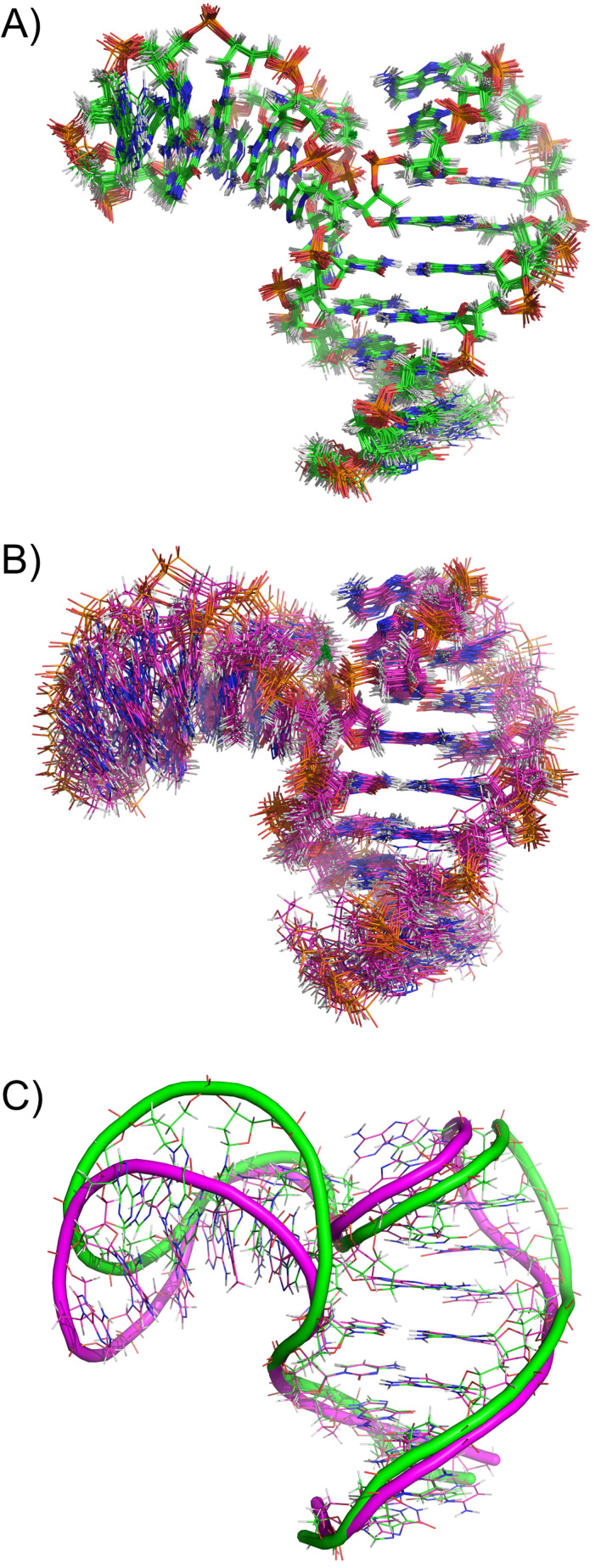
The effect of PCS data on the NMR structure of LnA_28. A) The structural bundle obtained using all data (including PCS), B) The structural bundle obtained excluding PCS from the dataset, C) Representative conformers from the two bundles superimposed (aligned by Helix 1).

However, what is the effect of the paramagnetic PCS restraints on the solved structure? Is their use actually beneficial? The most straightforward way to address this question is to repeat the structure calculations, excluding the PCS restraints from the experimental dataset. When such a set of calculations was performed it yielded an NMR structural bundle with a noticeably higher pairwise RMSD (1.51 Å) and also visually clearly less defined (Figure 8a vs. b). What is even more important is the fact that the interhelical angles between Helices I and II in the two sets of structures are actually different – 113°±2° and 99°±11° – for the structures calculated with and without PCS, respectively (Figure 8c). This finding can be related to the long‐recognized problem with structure determination based predominantly on short‐range NOE data. Due to the local nature of these restraints the global features of the biomolecule's fold – such as the exact mutual orientations of different helical elements – are often less precisely defined and their values in the final structures might be dictated more by the molecular dynamics force field than by the experimental data. Thus, the structural comparison just made provides a good demonstration of the need for long‐range structural restraints even in structure determination of relatively small biomolecules and the capability of paramagnetic effects to provide such restraints.

One other important feature that has to be addressed are the magnitudes of the **Δχ** tensors determined for the four paramagnetic lanthanides, collected in Table 1. The magnitude of **Δχ** is a characteristic dependent on the identity of the lanthanide ion, as well as, on its coordination geometry. Good reference points for the values that can be expected for each lanthanide ion in a sufficiently asymmetric binding site are provided by the **Δχ** tensors measured for the entire lanthanide ion series bound to either the protein calbindin D_9k_ ^[53]^ or to an inorganic lanthanide chelate Ln‐DOTA‐M7FPy,^[54]^ also provided here in Table 1. It is clear that the tensor magnitudes measured for lanthanide ions bound to LnA_28 are appreciably smaller than those reported for both reference systems. The lower‐than‐expected magnitudes of **Δχ** tensors are often related to motional averaging of the PCS data by large scale mobility between the metal and the biomolecule, however given how well the current PCS datasets fit to tensors this explanation seems unlikely in the case of LnA_28. Instead, in the current case the reduced tensor magnitudes may be explained by the lack of rigidity within the first coordination sphere of the lanthanide itself, with possible equilibria between different coordination geometries – e. g. with coordination numbers of 8 and 9 – suggested for the paramagnetic lanthanides bound to LnA_28 by the molecular simulations discussed above. The different coordination geometries would produce tensors of appreciably different orientations and the averaging between them could produce the observed effect of a smaller average tensor being measured experimentally. The reduced magnitude of **Δχ** tensors translate of course into shorter distances over which appreciable PCS can be measured, however with the **Δχ** values measured for LnA the range of PCS restraints remain significant. For the Tm^3+^ sample, PCS>0.1 ppm could still be observed for the most remote residue in the molecule (G28) at around 25 Å from the metal center and the high rigidity of the system signifies that even smaller PCS values should still remain interpretable in terms of structural parameters. While not measured in the current study lanthanide ions bound to LnA also have the potential to induce self‐alignment RDC. Taking the Tm^3+^ **Δχ** tensors magnitude from Table 1 one would expect RDCs of up to ∼5 Hz at a 700 MHz spectrometer or up to ∼10 Hz at 1000 MHz. While several‐fold smaller than what is provided by rigid tags in protein systems RDC of such magnitude can be reliably measured and used as additional global structural restraints in future NMR studies.

**Figure 9:**
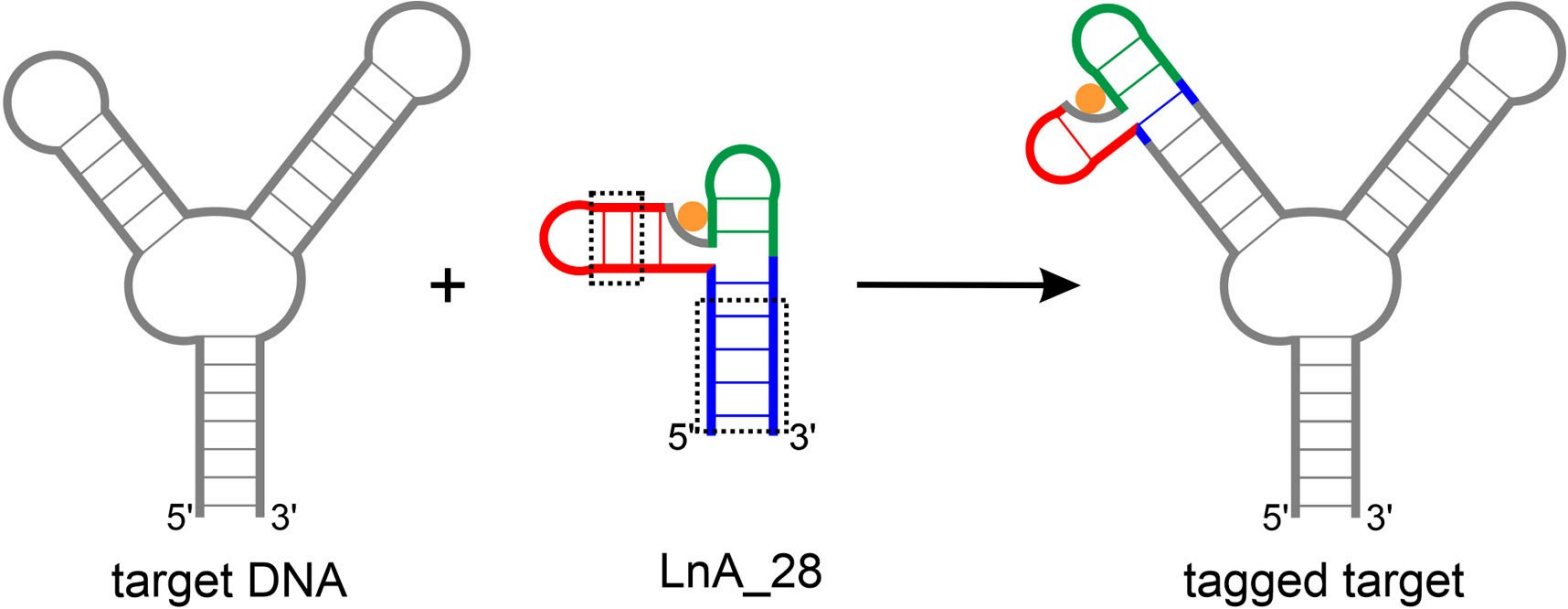
An example paramagnetic tagging approach using a LnA_28‐derived tag. Black dotted frames – residues from LnA_28 that can likely be omitted when turning the system into a paramagnetic tag

**On the prospect of transplantation of the lanthanide binding site into arbitrary DNA targets**. The LnA_28 construct has demonstrated its usefulness for measuring high quality PCS data in a DNA system, that are clearly interpretable in terms of molecular structure. The introduction of the lanthanide binding site found in this molecule into other DNA targets should thus, in principle, enable the study of their structures and dynamics by paramagnetic NMR. However, the size of the LnA_28 construct (∼8.5 kDa) would make it rather impractical to use as such a paramagnetic tag in its full form. Thus, the identification and extraction of a minimal lanthanide binding motif would be highly beneficial. The high‐resolution structure solved in this study situates the lanthanide binding site in a very intricate structural environment, unlikely to be recreated by just extracting a short stretch of residues directly interacting with Ln^3+^ from LnA_28. Nevertheless, as this study has demonstrated, the lanthanide binding capability is independent on the sequences of Helices I and II and these elements are only needed as structural support for the binding site. Thus, shorter variants of the paramagnetic tag can likely be derived from LnA_28 by substituting the residues forming one of the helices with a helical element already present in the DNA one wish to study (Figure 9) This general plan leaves several questions open, such as: which helix (I, II or III) is the best to be substituted, can the remaining helices in such fused constructs be further shortened (Figure 9, black box), how much “spacer” sequence has to be retained between the paramagnetic tag and structurally/functionally significant elements in target nucleic acid etc. A comprehensive study is now underway in our laboratory to clarify these issues and evaluate the range of practical usefulness of LnA‐derived constructs as paramagnetic tags for NMR spectroscopy.

## Conclusions

The results presented here demonstrate the feasibility of inducing high‐quality PCS for structural studies of nucleic acid systems using intrinsic lanthanide binding sites. While the site studied here turned out to be rather structurally intricate it has the potential to yield a useful paramagnetic tag. It also constitutes a proof‐of‐concept to justify the search for other similar sites through additional SELEX experiments. Compared to paramagnetic tagging methods relying on inorganic chelates, also currently under development for nucleic acids,[^20^, ^21^, ^22^] tags constructed around intrinsic sites would likely add a higher molecular‐weight burden to their targets, as well as, could potentially suffer reduced **Δχ** tensor magnitudes. On the other hand, using intrinsic sites for paramagnetic tagging is much more compatible with isotopic labeling of the target^[55]^ and, as demonstrated here, can provide exceptionally rigid tags.

## Experimental Section

**Sample preparation**: All the DNA constructs were purchased from Metabion GmbH. Before usage, they were purified from residual salt (remaining after HPLC) by centrifugation on Amicon filters in the same buffer used during subsequent experiments. For all spectral measurements, including NMR, CD and UV spectroscopies, 10 mM cacodylate buffer (pH 6.25) containing 100 mM NaCl was used. All the NMR sample concentrations were around 700 μM and the final DNA‐lanthanide complexes were obtained by titrating the appropriate metals into the DNA solution, already in the final buffer conditions.

**UV melting**: Thermal denaturation curves at 260 nm were recorded with a JASCO V‐750 spectrophotometer with a water cooled thermoprogrammer, using 5 mm (150 μL) quartz microcuvettes. The samples were protected against evaporation by the silicone oil. The temperature range was 10–88 °C, using the scan rate 0.2 °C/min. The melting curves were normalized and plotted using in‐house Python scripts.

**CD spectroscopy**: The circular dichroism spectra (CD) were recorded using a JASCO J815 spectropolarimeter equipped with a Peltier temperature controller. Cuvettes with a path length of 0.5 cm were used (sample volumes 1500 μl). Spectra were collected in the range between 215 and 330 nm, as a sum of seven repetitions at 25 °C and the buffer baseline was subtracted from each spectrum. The sample concentrations were 7.6 and 6.4 μM for LnA_28 and the original construct, respectively. CD spectra were expressed in the units of molar ellipticity Δε [cm^2^ mmol^−1^], without normalization by the number of residues in the molecule.

**NMR spectroscopy**: All NMR spectra were collected using a 700 MHz Bruker Avance III spectrometer equipped with a QCI‐P CryoProbe. The resonance assignment of non‐exchangeable protons and phosphorus atoms in each of the five samples (containing Lu^3+^, Eu^3+^, Yb^3+^, Ce^3+^ and Tm^3+^) was achieved using standard procedures,[^56^, ^57^] through the analysis of NOESY, TOCSY, HC‐HSQC and HP‐COSY spectra recorded in 100 % D_2_O at 25 °C and 45 °C. The exchangeable protons were assigned using NOESY spectra measured in 90 % H_2_O / 10 % D_2_O at 25 °C. All the spectra were analyzed in NMRFAM‐Sparky.^[58]^

**Restraint generation and NMR structure calculations**: Three types of NMR restraints were employed in the structure calculations for LnA_28: NOE‐derived distance restraints, dihedral angle restraints and pseudocontact shift (PCS) restraints. The distance restraints between non‐exchangeable protons were extracted from the peak volumes in NOESY spectra recorded in D_2_O with and 150 ms mixing time. The NOESY cross‐peaks were classified as strong (1.8–3.0 Å), medium (2.0–4.0 Å) and weak (2.2–5.0 Å), using fixed distances (H5‐H6 in cytidines and H2’‐H2’’ in deoxyriboses) as reference. The distance restraints between exchangeable protons were derived from the NOESY spectra recorded in H_2_O (150 ms mixing) using a similar procedure, with the mean volume of the imino‐amino contacts of guanosines used as reference.

Regarding dihedral angle restraints, the backbone dihedral angles α and ζ were restrained to exclude the trans rotamer for all residues with ^31^P chemical shift within the standard range. Moreover, for residues for which the P(n)‐H4’(n‐1) cross peak was observable the β and γ dihedral angles were restrained to 180° and 60°, respectively (±60°). The χ dihedral angle was in turn restrained to the *anti* orientation for all residues except C16 for which it was restrained to *syn*, based on the intensities of H1′‐H6/H8 NOESY cross‐peak.

The PCS sets for each paramagnetic metal were calculated as chemical shift differences between the appropriate paramagnetic sample and the diamagnetic reference (Lu^3+^ sample) for each proton that was assignable in both samples. The PCS experienced by a given nucleus depends both on the metal‐nucleus distance and the orientation of the metal‐nucleus vector in the molecular frame of reference according the following formula:[^3^, ^4^]

(1) $$\delta^{PC}=\frac{1}{12\pi }\frac{1}{r^{3}}\left[\Delta \chi_{ax}\left(3\cos^{2}\theta -1\right)+\frac{3}{2}\Delta \chi_{rh}\sin^{2}\theta \cos2\Omega \right]$$

Where: $\delta^{PC}$ is the pseudo‐contact contribution to the chemical shift (PCS), $\Delta \chi_{ax}$ and $\Delta \chi_{rh}$ are the axial and rhombic components of the magnetic susceptibility anisotropy tensor **Δχ**, r is the electron‐nucleus distance, while θ and Ω are the polar coordinates of the electron‐nucleus vector in the principal axis frame (PAF) of **Δχ**.

The structure calculations were performed using a two‐step procedure, involving 1) initial folding with diamagnetic restraints including NOE and dihedral angles and 2) refinement using both diamagnetic and paramagnetic (PCS) NMR data. Both stages were executed in the SANDER module of the AMBER 18 molecular dynamics suite of programs.^[34]^ The first step was performed using an implicit solvent model (Generalized Born) without including a metal ion in the simulation box, while the second step employed an explicit description of the solvent to correctly model a potentially partially hydrated Ln^3+^ ion. The specifics of the force fields used are provided in the ‘MD simulations’ section below. In order to obtain a first approximation of the **Δχ** tensors, as well as the position of the metal ion, the PCS data was fitted (Eq. (1)) to the preliminarily folded structure (after step 1) using the program FANTEN.^[59]^ The obtained paramagnetic center coordinates were used to place the metal ion in the preliminarily folded structure, for the 2^nd^ step of calculations involving PCS. The **Δχ** tensor parameters obtained from the first PCS fitting are not necessarily optimal ones ‐ as they are determined from the fit of the experimental data to a molecular structure (according to Equation (1)), a reasonable, but non‐ideal, structure will yield reasonable, but non‐optimal tensor parameters. Most software used to calculate biomolecular structures (including SANDER) do not have the capability to further optimize the tensor parameters during the run and thus an iterative approach to structure determination using PCS is most often applied. It consists of first performing the structure calculations using **Δχ** parameters from the preliminary fit, then recalculating the tensor using the resulting improved structures and repeating these steps until achieving convergence of both the structure and **Δχ** parameters. In the current case five rounds of tensor optimization were needed to achieve convergence evaluated using criteria proposed in,^[60]^ calculating 50 structures at each step. In the final step 100 structures were calculated instead, using the converged **Δχ** tensors and then 20 were selected based on their consistency with both the diamagnetic and paramagnetic NMR data.

Regarding the metal ion description during structure calculations, force field parameters for Lu^3+^ were used throughout the calculations. To verify that this choice did not significantly influence the final structures the last step of calculations was repeated using parameters for La^3+^ instead. A structural bundle composed of 10 best structures from the Lu^3+^ calculations and 10 best structures calculated with La^3+^ displayed the mean pairwise RMSD of 0.72, practically identical to that of the all‐Lu^3+^ bundle.

**MD simulations**: All MD simulations were performed using AMBER18 using the parm99bsc0χ_OL15_ force field^[61]^ with SPC/E water model and Joung‐Cheatham parameters for monovalent cations^[62]^ and Li‐Merz parameters for lanthanide ions.^[63]^ The Li‐Merz parameters were used in their variant optimized to best reproduce the experimental Ion‐Oxygen Distance (IOD) in hydrated ions, which is the default (recommended) parameter set as of AMBER18. The first conformer from the NMR bundle was solvated using octahedral box of SPC/E water with a minimum distance between box walls and solute of 10 Å and additional Na^+^ ions were added to neutralize the system. The solvated system was minimized and equilibrated. The long‐range electrostatics were calculated using the particle mesh Ewald method with the nonbonded cutoff set to 9 Å. The covalent bonds were constrained using SHAKE and the integration time step was set to 2 fs. The Langevin thermostat with collision frequency 2.0 ps^−1^ was used to control the temperature and Berendsen barostat for constant pressure simulation.

The water density maps around the average MD structures (Figure S3) were calculated using the CPPTRAJ^[64]^ ‘grid’ routine and visualized using UCSF‐Chimera.^[65]^ The hydrogen bond occupancies in the simulation were calculated using CPPTRAJ ‘hbond’ routine with standard cutoff parameters.

## Conflict of interest

The authors declare no conflict of interest.

1

## Supporting information

As a service to our authors and readers, this journal provides supporting information supplied by the authors. Such materials are peer reviewed and may be re‐organized for online delivery, but are not copy‐edited or typeset. Technical support issues arising from supporting information (other than missing files) should be addressed to the authors.

Supporting Information

## Data Availability

Atomic coordinates and the list of experimental restraints for the reported NMR structure have been deposited with the Protein Data bank under accession number 7QB3 while the chemical shifts have been deposited at the BMRB under the number 34685.
