# Supplementary material for: Solution Structure of a Lanthanide‐binding DNA Aptamer Determined Using High Quality pseudocontact shift restraints
Source: Chemistry. 2022 Oct 1;28(66):e202202114. doi: 10.1002/chem.202202114 (PMC9828363; doi:10.1002/chem.202202114)
Supplement: Supplementary file 1 — Supporting Information [file CHEM-28-0-s001.pdf]

Chemistry–A European Journal

Supporting Information

Solution Structure of a Lanthanide-binding DNA Aptamer Determined Using High Quality pseudocontact shift restraints

Witold Andrałojć,* Julia Wieruszewska, Karol Pasternak, and Zofia Gdaniec

Table of contents:

- Supplementary results and discussion 3
- Figure S1. CD spectroscopy and UV-melting analysis 8
- Figure S2. CD titrations of LnA_28 with Ce^{3+} and Lu^{3+} 9
- Figure S3. NOESY spectra Eu^{3+} and Lu^{3+} 10
- Figure S4. NOESY spectra for the five metals superimposed. 11
- Figure S5. Water molecule “density map” in the MD simulations 12
- Figure S6. The fitting of ^{31}P PCS to the structure of LnA_28 13
- Table S1. The DNA constructs of LnA 14
- Table S2. ^{1}H and ^{31}P chemical shifts 15
- Table S3. Structure determination statistics for LnA_28 22
- Table S4 Occupancies of hydrogen bonds in MD simulations 23

Supplementary results and discussion

Sequence optimization

The general goal of the performed sequence optimization was to obtain a significantly shortened construct of the aptamer, while conserving unperturbed lanthanide-binding capability. The design of the shortened constructs was based on the assumption that the general secondary structure of each LnA variant should remain the same as presented in Figure 1a (Helix I - 9 to 13 nt loop - Helix II), but that the identity of the residues forming the two helices can be freely altered (with the exception of perhaps the two base pairs directly adjacent to the loop region). The actual sequences of the shortened helices were chosen to ensure both their highest thermodynamic stability, as well as the preference of the construct for only a single folded structure. Both the thermodynamic stability and the existence of alternative secondary structures was evaluated in silico using the program UnaFold [12]. The loop region, on the other hand, is the most likely location of the lanthanide binding site (given that its 5'-terminal part – CCGC - is the only conserved stretch of non-paired bases among the constructs obtained in[13]) and thus only its central part (3-8 residue variable region) was modified during the optimization. Even this limited intervention was not done by freely mutating residues within this region, but instead by using the sequences found in the different constructs from the original study. This set of assumptions regarding the allowed range of sequential freedom turned out to be very reasonable, as all but one among the designed constructs retained unaltered lanthanide-binding capability (see Table S1), as monitored using 1D NMR, UV-melting and /or CD spectroscopy. The only exception was a construct in which Helix I was reduced to 4 bp, which likely made it too thermodynamically unstable. This led us to the conclusion that at least 5 bp Helix 1 one is necessary for the proper folding. Regarding Helix 2, we were able to observe unaltered function even for constructs in which it was 3 bp long and capped with a GAA triloop. For the loop region, its shortest variant in the original study was 9 nt long and we have found that combining this loop sequence with our shortened helices also gave rise to no alteration of lanthanide binding. Thus, the final construct to be used in the NMR study was composed of 28 nt (no. 7 in Table S1; 5 bp Helix 1, 3 bp Helix 2, GAA triloop, 9 nt lanthanide binding loop)

CD spectroscopy and UV-melting analysis

Figures S1a and b present the CD spectral response observed upon Eu^{3+} titration to the original LnA (missing the single-stranded 5' overhang) and the shortened LnA_28 constructs, respectively. In both cases already the metal-free samples (blue curves) demonstrate CD profiles typical for B-DNA, with ellipticity maxima around 280 nm, suggesting that the expected helical

elements form even without the presence of the metal. Both molecules, however, also display progressive spectral changes with increasing Eu^{3+} concentration, most pronounced in the 235-255 nm range. The transitions saturate between 1.0 and 1.5 equivalents of Eu^{3+} in each case and the resulting binding curves can be fitted using a 1:1 interaction model, yielding K_d values in the low-hundred nanomolar range (~100-300 nM depending on the wavelength selected for fitting; Figure S1c-d), consistent with the original paper. In case of the original construct, however, a second transition actually begins to be observable after the first one is saturated (spectral changes around 280 nm saturate around two equivalents and then restart roughly above four), likely corresponding to lanthanide binding to a second much weaker site. Lanthanide ions are actually well known to interact with bases in single stranded regions of DNA without much sequence specificity and thus the observation of such secondary interactions is not surprising. On the other hand, the lack of such interactions for the shortened construct might suggest that its apparent single-stranded region is in reality well-structured, with nucleobases involved in pairing interactions. CD titrations with two different lanthanide ions (Ce^{3+} and Lu^{3+}) were also performed, yielding qualitatively the same behavior (Figure S2) as well as very similar estimates of K_d values, confirming the previous observation[13] that the aptamer is capable of interacting with the entire lanthanide ion series.

With the K_d values of the constructs confirmed, the metal-free and metal-saturated forms of the two aptamers were subjected to UV melting analysis (Figure S1e-f). In both cases the shape of the melting profile turned out to be greatly dependent on the metal binding. Namely, it changed from a multistep, non-cooperative melting over a broad range of temperatures when no metal is present, to a single well-defined melting transition when the metal binding is saturated. Such a behavior suggests that upon metal binding the molecule converts from a state in which several secondary structure elements are present, yet do not interact with each other, to one containing a well-defined tertiary structure, melting in a single transition. Interestingly, when the melting temperatures (T_m) of the bound aptamers are compared it turns out that, despite having significantly shortened Helices I and II, the LnA_28 construct is actually more thermally stable. Such a result might be related to the stabilizing effect of the GAA triloop capping Helix II, however it might as well stem from the different sequences of the loop regions.

AMBER PCS fitting routine modification/extension

A set of subroutines designed to introduce PCS restraints into NMR structural calculations in SANDER is already available[14] and shipped as part of the official AMBER software suite. However, its original version is not able to use PCS data originating from several different samples

concurrently, instead being able to handle a somewhat rarely explored case of several paramagnetic centers being present in the same sample. The source code of the PCS handling subroutines was thus modified to provide the missing functionality. Several ease-of-use features, as well as, bug corrections were also added in the process (listed below).

1) The input file for PCS fitting now requires the specification of the number of samples for which PCS were measured (‘nsam’). The possibility of having several paramagnetic centers in the same sample is also retained for legacy compatibility (with the original parameter name ‘nfe’), although not used in the current work (‘nfe’ set to 1). The ‘nsam’ and ‘nfe’ values are then used to assign the paramagnetic tensors specified in the file to the correct samples (if ‘nfe’=1, then simply tensor1 corresponds to sample1, tensor2 to sample2 and so forth). Each PCS in the input file is now also labelled with a parameter ‘idsam’, denoting the number of sample for which it was measured, allowing to link it with the correct paramagnetic tensor during the calculations. As one atom can now experience forces related to several different PCS values (if a PCS value was measured for it for more than one sample) the net force acting on that atom due to the PCS term (returned by the subroutine to the main SANDER program) is now calculated as a vector sum of the forces due to individual PCS.

2) While analyzing the source code it was noticed that in the original implementation the force acting on an atom due to the PCS term was proportional not only to the PCS violation (the difference between the experimentally measured PCS and its value calculated using current atom coordinates) as intended, but also to the absolute value of the PCS function gradient at that point in space (the PCS function gradient used to determine the direction of the force to be applied was not normalized). Thus, while the direction of the applied force was correct, its absolute value not necessarily reflected the magnitude of the violation of the given PCS. To resolve this issue a normalization is now performed on the calculated PCS function gradient before using it to evaluate the force due to the PCS term.

3) The original set of subroutines was intended to be used together with a program named FNATASIAN[14], able to determine paramagnetic tensor parameters through the fit of the experimental PCS to molecular structures. Thus, both use the same Euler angle convention to define the Principal Axis Frames of the paramagnetic tensors. Unfortunately, the Euler angle convention used by FANTASIAN is not compatible with the newer and more popular paramagnetic tensor fitting software like FANTEN[5] or Numbat[15]. Thus, the set of equations used to transform the Euler angle values defining the tensors into rotation matrices was modified to comply with the szyz Euler angle convention (the default convention of FANTEN).

4) Some additional quality-of-life changes were also made. Namely: the paramagnetic centers are now identified by their atom numbers in the PDB file instead of atom names (which might be ambiguous) and the PCS input file is now checked for the consistency of the atom multiplicity provided by the user. The possibility of applying the PCS-related force to paramagnetic metal ion (with the same magnitude and opposite direction with respect to the force experienced by the atom for which the PCS was measured) was also added.

The modified PCS fitting routine was tested using both experimental and simulated PCS data, producing expected behavior. The modified routine source code is available from the authors upon request.

References

[1] G. Varani, F. Aboul-ela, F. H.-T. Allain, *Prog. Nucl. Magn. Reson. Spectrosc.* **1996**, *29*, 51–127.

[2] S. S. Wijmenga, B. N. M. van Buuren, *Prog. Nucl. Magn. Reson. Spectrosc.* **1998**, *32*, 287–387.

[3] W. Lee, M. Tonelli, J. L. Markley, *Bioinformatics* **2015**, *31*, 1325–7.

[4] D. A. Case, D. S. Cerutti, T. E. I. Cheatham, T. A. Darden, R. E. Duke, T. J. Giese, H. Gohlke, A. W. Goetz, D. Greene, N. Homeyer, S. Izadi, A. Kovalenko, T. S. Lee, S. LeGrand, P. Li, C. Lin, J. Liu, T. Luchko, R. Luo, D. Mermelstein, K. M. Merz, G. Monard, H. Nguyen, I. Omelyan, A. Onufriev, F. Pan, R. Qi, D. R. Roe, A. Roitberg, C. Sagui, C. L. Simmerling, W. M. Botello-Smith, J. Swails, R. C. Walker, J. Wang, R. M. Wolf, X. Wu, L. Xiao, D. M. York, P. A. Kollman, **2017**, AMBER 2017, University of California, San Francisc.

[5] M. Rinaldelli, A. Carlon, E. Ravera, G. Parigi, C. Luchinat, *J. Biomol. NMR* **2014**, *61*, 21–34.

[6] L. Russo, M. Maestre-Martinez, S. Wolff, S. Becker, C. Griesinger, *J. Am. Chem. Soc.* **2013**, *135*, 17111–17120.

[7] M. Zgarbová, M. Otyepka, J. Šponer, A. Mládek, P. Banáš, T. E. Cheatham, P. Jurečka, *J. Chem. Theory Comput.* **2011**, *7*, 2886–2902.

[8] I. S. Joung, T. E. Cheatham, *J. Phys. Chem. B* **2008**, *112*, 9020–9041.

[9] P. Li, L. F. Song, K. M. Merz, *J. Phys. Chem. B* **2015**, *119*, 883–895.

[10] D. R. Roe, T. E. Cheatham, *J. Chem. Theory Comput.* **2013**, *9*, 3084–3095.

[11] E. F. Pettersen, T. D. Goddard, C. C. Huang, G. S. Couch, D. M. Greenblatt, E. C. Meng, T. E. Ferrin, *J. Comput. Chem.* **2004**, *25*, 1605–1612.

[12] M. Zuker, *Nucleic Acids Res.* **2003**, *31*, 3406–3415.

[13] O. Edogun, N. H. Nguyen, M. Halim, *Anal. Bioanal. Chem.* **2016**, *408*, 4121–4131.

[14] L. Banci, I. Bertini, G. G. Savellini, A. Romagnoli, P. Turano, C. Cremonini, M.A., Luchinat, H. B. Gray, *Proteins* **1997**, *29*, 68–76.

[15] C. Schmitz, M. J. Stanton-Cook, X.-C. Su, G. Otting, T. Huber, *J. Biomol. NMR 2008 413* **2008**, *41*, 179–189.

Figure S1. CD spectroscopy and UV-melting analysis of the original and shortened constructs of LnA, A-B) CD spectral changes upon Eu^{3+} titration, C-D) K_d estimation from CD tirations, E-F) UV-melting profile for the free and ligand-saturated forms of the aptamer.

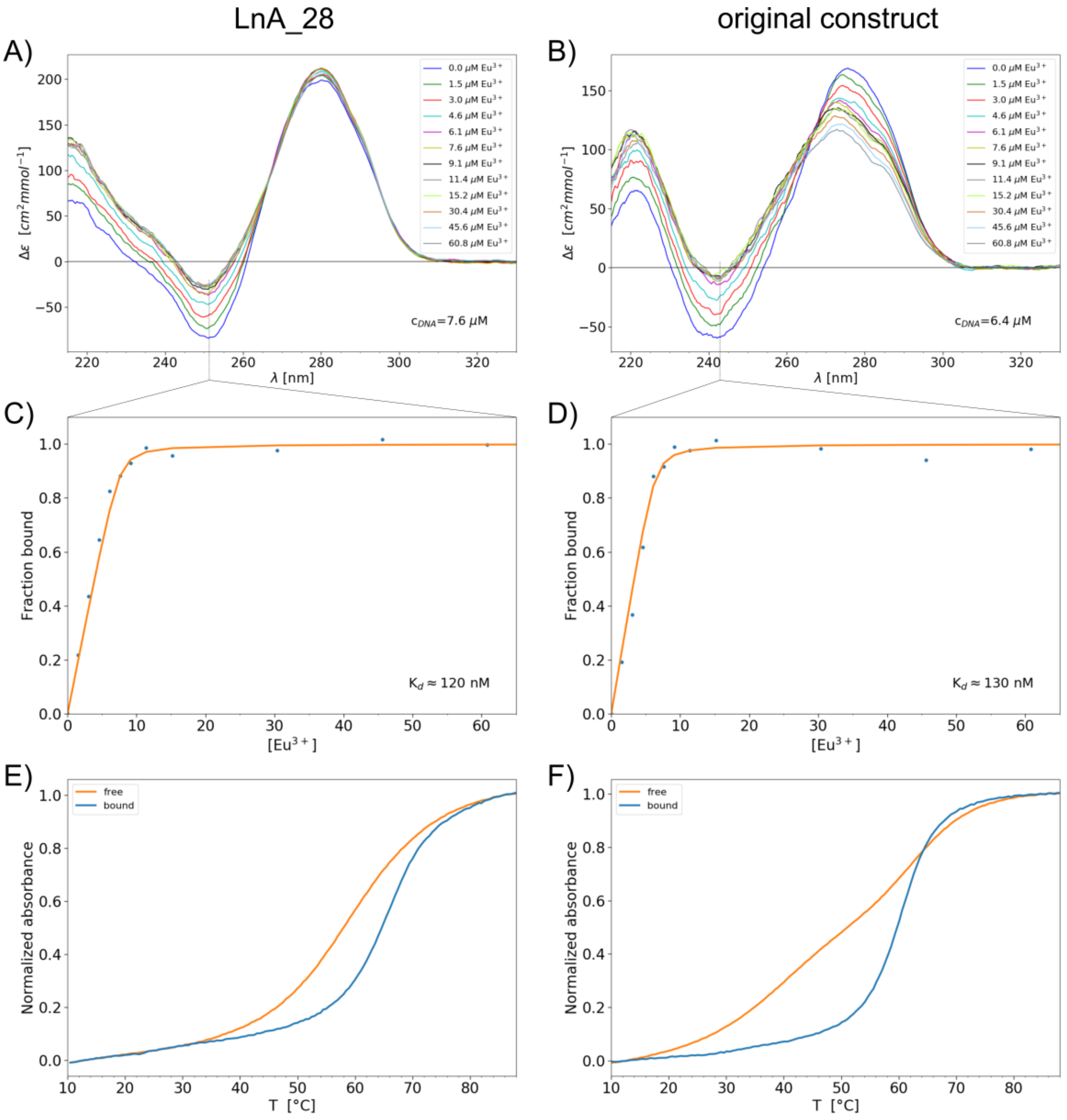

Figure S2. CD titrations of LnA_28 with Ce^{3+} (A) and Lu^{3+} (C) and of the original construct with the same metals (B and D).

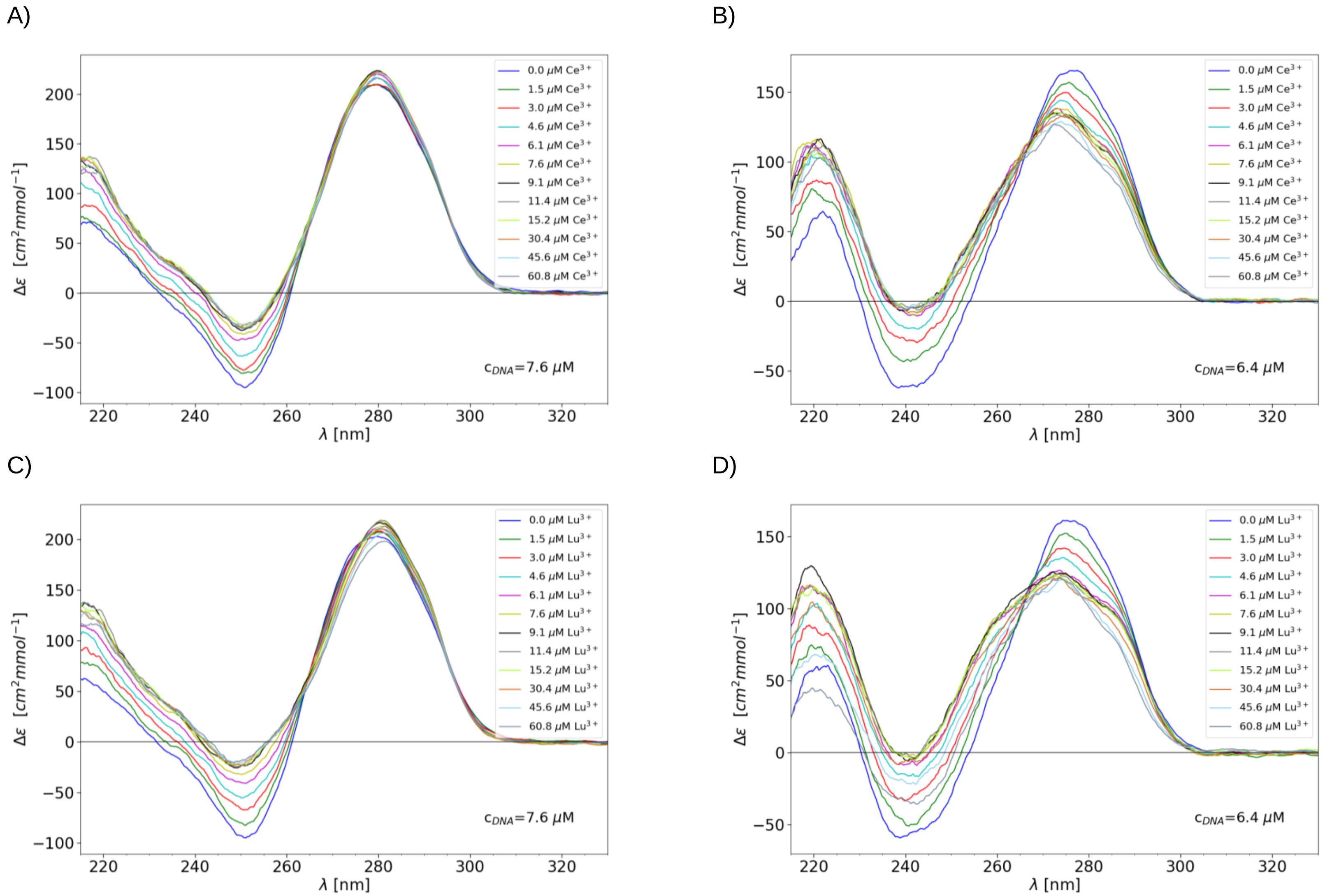

Figure S3. Aromatic-to-anomeric region of the NOESY spectra recorded in D_2O for (A) Lu^{3+} and (B) Eu^{3+}. The path of sequential connectivities ('NOESY walk') is marked in each spectrum with lines color-coded: Helix 1 – blue, Helix 2 – red, Helix 3 – green.

A)

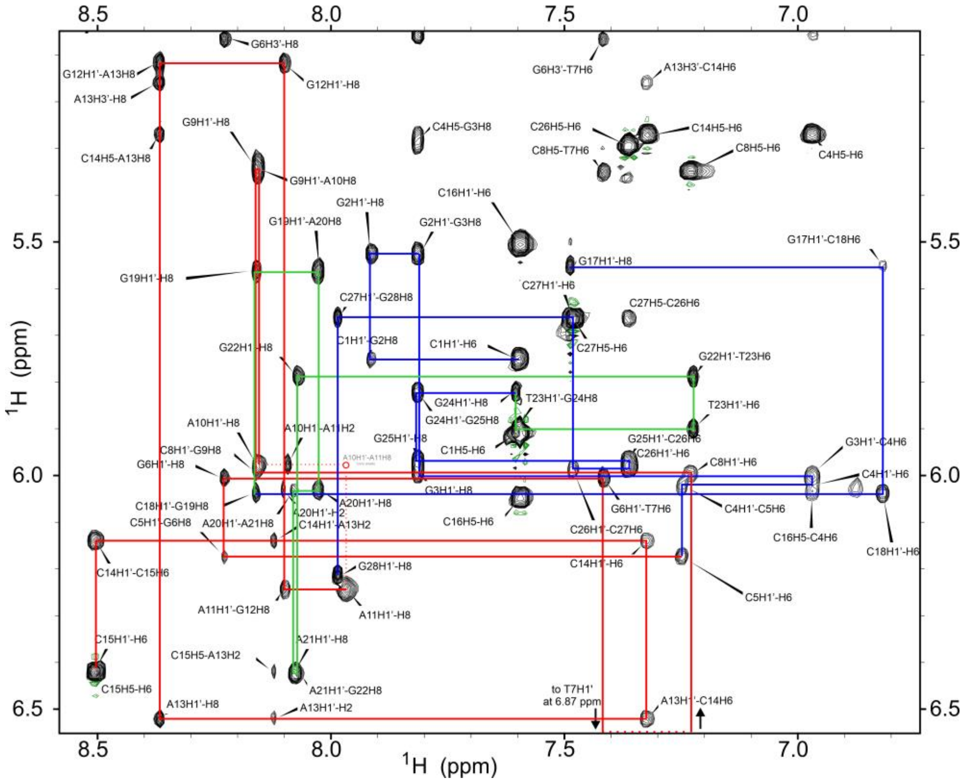

B)

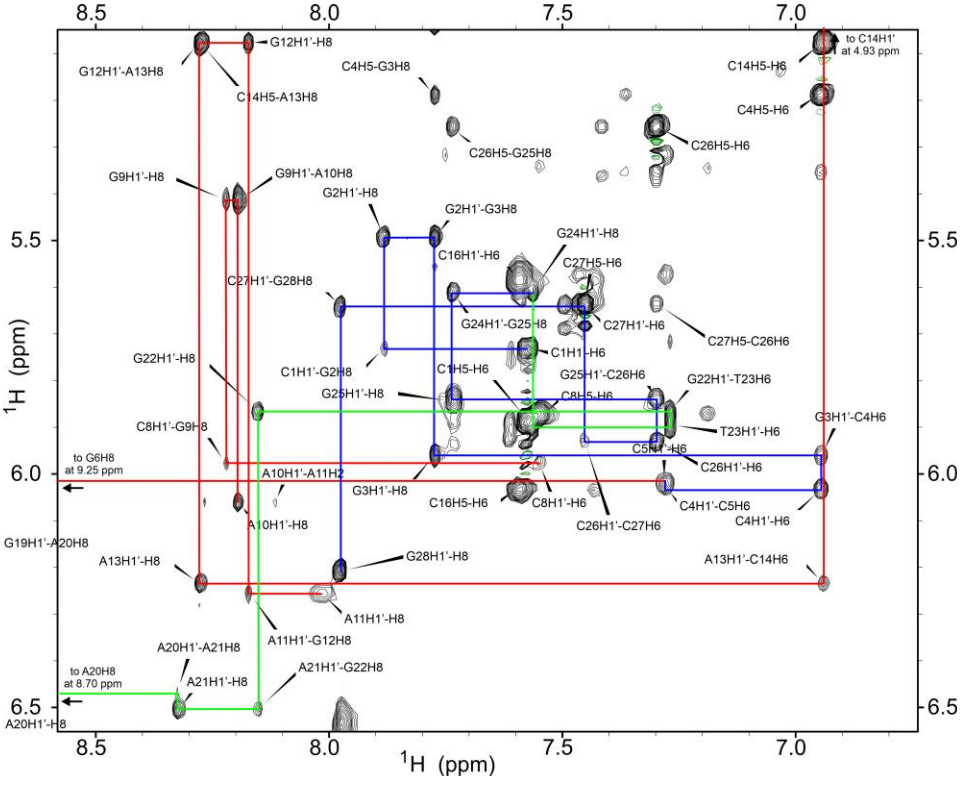

Figure S4. Aromatic-to-anomeric regions of the NOESY spectra recorded in D_2O for the five metals superimposed. The spectra are color coded: Lu^{3+} – black, Eu^{3+} – blue, Yb^{3+} – red, Ce^{3+} – green, Tm^{3+} – yellow. For two selected cross-peaks the resonance shifts (PCS) between the diamagnetic and the four paramagnetic samples are marked with magenta arrows.

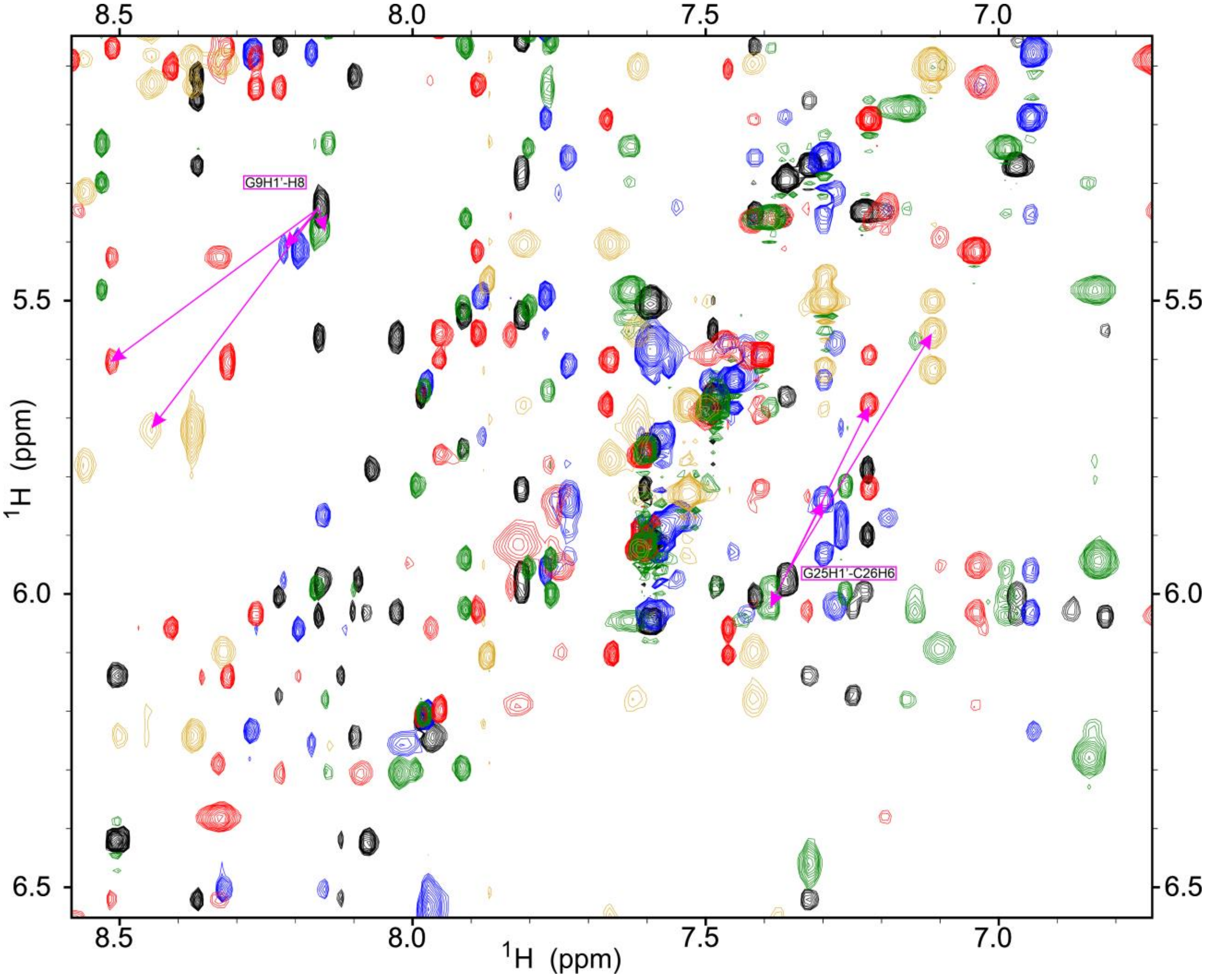

Figure S5. Water molecule “density map” in the MD simulations performed in the presence of Lu^{3+} (A) and La^{3+} (B). The regions of space frequently occupied the water oxygen atoms are colored in magenta, while the ones containing water hydrogens in green.

A)

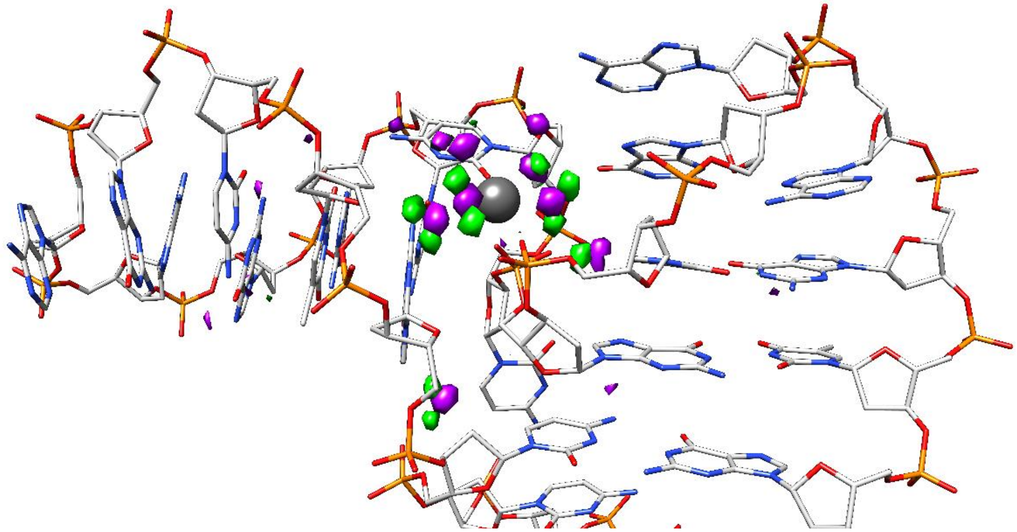

B)

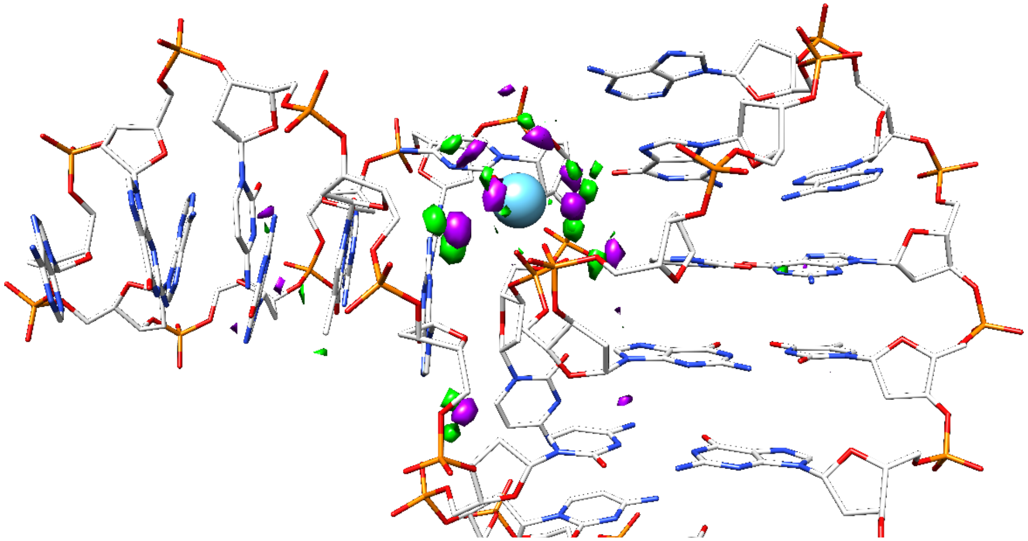

Figure S6. The fitting of ^{31}P PCS to the structure of LnA_28 using **Δχ** tensor parameters derived for ^{1}H PCS.

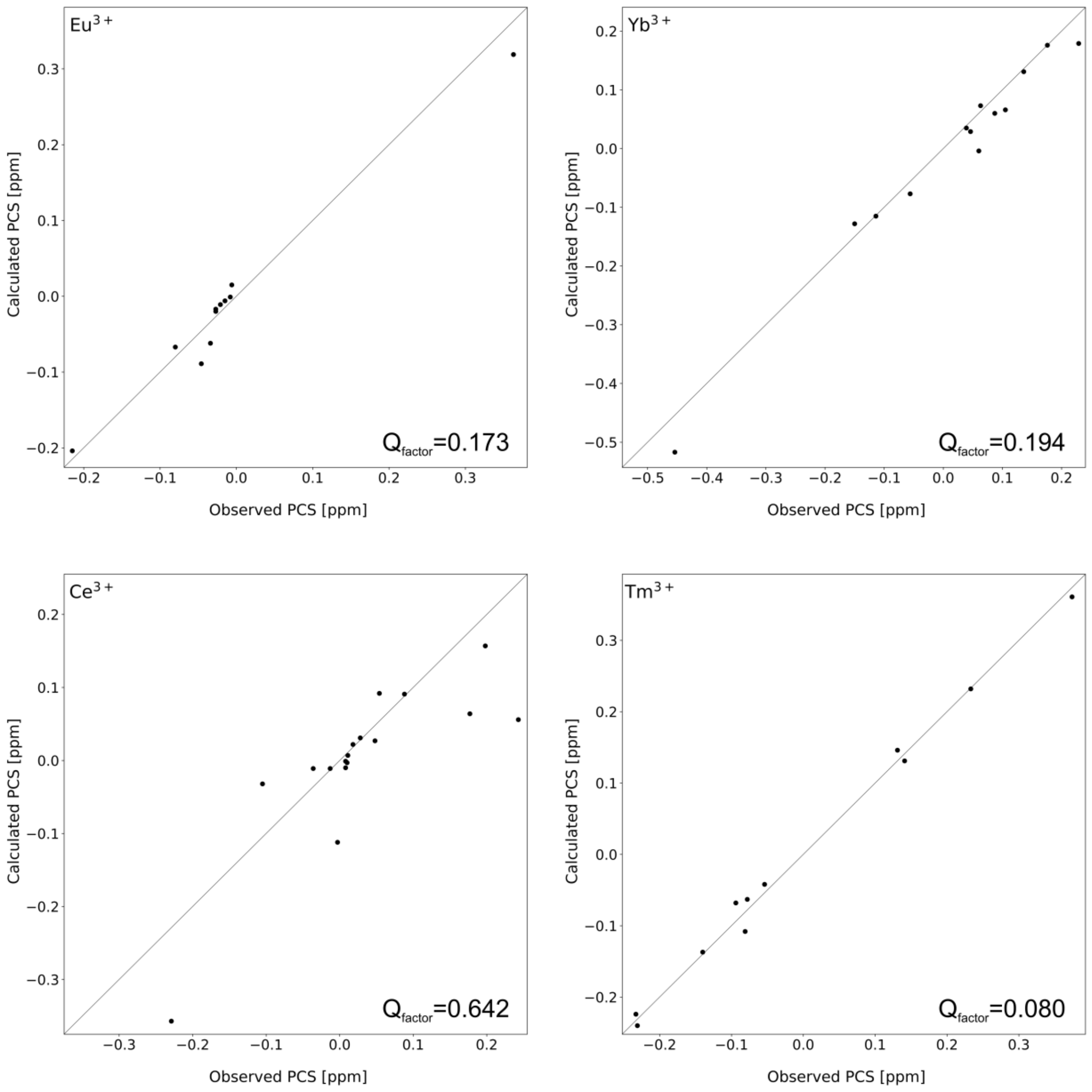

Table S1. The DNA constructs tested in the scope of sequence optimization of LnA for NMR study.

No.	sequence	sequence length	Ln^{3+} ion binding[a]	Stem 1	Stem-loop 2	Ln-binding loop	Comment
1	5'- AGGCTCTCGGGACGACCAGTTG GTCCCGCTTTATGTGTCCCGAG -3'	44 nt	OK	8 bp	4bp+4nt	11 nt	the aptamer designed in [13]
2	5'- CTCGGGACGACCAGTTG GTCCCGCTTTATGTGTCCCGAG -3'	39 nt	OK	8 bp	4bp+4nt	11 nt	as no. 1, without the 5 nt 5'-overhang
3	5'- CGGCACGACCGAAGGTCCCGC TTTATGTGTGCCG -3'	34 nt	OK	6 bp	4bp+3nt	11 nt	as no. 2, redesigned Helix 1, Helix 2 capped with GAA
4	5'- CGGCCGTCGAAGACCCGC TTTATGTGGCCG -3'	30 nt	OK	5 bp	3bp+3nt	11 nt	as no. 3, further redesigned Helices 1 and 2
5	5'- GCGCGTCGAAGACCCGC TTTATGTGCGC -3'	28 nt	perturbed	4 bp	3bp+3nt	11 nt	as no. 4, further shortened Helix 1 (too much)
6	5'- CGGCACGACCGAAGGTCCCGC GTAAGTGTGCCG -3'	33 nt	OK	6 bp	4bp+3nt	10 nt	as no. 3, loop sequence of construct 1 in [13]
7	5'- CGGCACGACCGAAGGTCCCGC GAAGTGTGCCG -3'	32 nt	OK	6 bp	4bp+3nt	9 nt	as no. 3, loop sequence of construct 21 in [13]
8	5'- CGGCCGTCGAAGACCCGC GAAGTGGCCG -3'	28 nt	OK	5 bp	3bp+3nt	9 nt	as no. 4, loop sequence of construct 21 in [13]

[a]as evaluated by NMR spectroscopy and/or UV melting experiments (see main text)

Table S2. ^{1}H and ^{31}P chemical shifts measured for LnA_28 in complex with five different lanthanide ions (Lu^{3+}, Eu^{3+}, Yb^{3+}, Ce^{3+}, Tm^{3+})

Residue	Atom	Lu	Eu	Yb	Ce	Tm
C1	H1'	5.752	5.732	5.764	5.755	5.680
C1	H2'	1.859	1.839	1.880	1.858	1.782
C1	H2''	2.358	2.336	2.383	2.361	2.282
C1	H3'	4.685	4.667	4.711	4.685	4.633
C1	H4'	4.061	4.048	4.077	---	---
C1	H5'	3.699	3.684	---	---	---
C1	H5''	---	---	---	---	---
C1	H5	5.911	5.885	5.906	5.918	5.829
C1	H6	7.598	7.574	7.607	7.601	7.529
G2	H1'	5.526	5.492	5.557	5.513	5.405
G2	H2'	2.692	---	2.751	2.685	2.609
G2	H2''	2.745	---	2.800	2.732	2.632
G2	H3'	4.978	4.954	5.034	4.969	4.915
G2	H4'	4.313	4.293	4.346	4.304	4.244
G2	H5'	4.076	---	---	---	---
G2	H5''	3.966	3.949	3.997	---	---
G2	P	-4.475	-4.496	-4.436	-4.467	-4.530
G2	H8	7.915	7.882	7.953	7.915	7.810
G3	H1'	5.995	5.958	6.029	5.957	5.771
G3	H2'	2.598	2.571	2.680	2.598	2.444
G3	H2''	2.701	2.649	2.780	2.653	2.488
G3	H3'	5.058	5.037	5.132	5.043	4.965
G3	H4'	4.473	4.457	4.512	4.455	4.362
G3	H5'	---	---	---	---	---
G3	H5''	4.160	4.139	---	---	---
G3	P	-4.374	-4.401	-4.312	-4.364	-4.452
G3	H8	7.812	7.773	7.891	7.803	7.662
C4	H1'	6.021	6.028	5.951	5.994	5.392
C4	H2'	1.363	1.382	1.404	1.372	0.766
C4	H2''	2.354	2.395	2.339	2.269	1.525
C4	H3'	4.843	4.912	4.914	4.817	4.527
C4	H4'	4.242	4.274	4.239	---	---
C4	H5'	---	---	---	---	---
C4	H5''	---	---	---	---	---
C4	P	-4.533	-4.549	-4.428	-4.569	-4.614
C4	H5	5.272	5.188	5.417	5.240	---
C4	H6	6.970	6.945	7.038	6.987	6.558

C5	H1'	6.173	6.016	5.390	6.027	---
C5	H2'	2.041	---	1.610	1.778	---
C5	H2''	1.747	---	0.632	1.292	---
C5	H3'	4.985	5.315	4.751	4.606	---
C5	H4'	4.171	---	3.843	---	---
C5	H5'	---	---	---	---	---
C5	H5''	---	---	---	---	---
C5	P	-4.841	---	-4.782	---	---
C5	H5	4.847	4.654	5.019	4.774	---
C5	H6	7.249	7.280	7.100	7.143	---
G6	H1'	6.005	---	---	---	---
G6	H2'	2.795	4.321	---	---	---
G6	H2''	2.891	5.256	---	---	---
G6	H3'	5.066	---	---	---	---
G6	H4'	4.483	---	---	---	---
G6	H5'	4.176	---	---	---	---
G6	H5''	4.058	---	---	---	---
G6	P	-5.154	-4.791	-5.608	-5.383	---
G6	H8	8.229	9.250	---	7.354	---
T7	H1'	6.875	---	---	---	---
T7	H2'	2.127	3.301	---	---	---
T7	H2''	2.551	3.138	---	---	---
T7	H3'	4.979	---	---	---	---
T7	H4'	4.474	---	---	---	---
T7	H5'	---	---	---	---	---
T7	H5''	---	---	---	---	---
T7	P	-4.816	---	---	---	---
T7	Me	1.403	2.481	---	0.844	---
T7	H6	7.417	9.427	---	6.462	---
C8	H1'	5.994	5.973	6.519	6.180	---
C8	H2'	1.870	1.981	2.665	1.865	---
C8	H2''	---	2.314	2.768	2.329	---
C8	H3'	4.870	---	5.425	4.926	---
C8	H4'	4.251	---	---	---	---
C8	H5'	4.138	---	---	---	---
C8	H5''	---	---	---	---	---
C8	P	-4.718	---	---	---	-4.687
C8	H5	5.350	5.874	6.382	5.173	---
C8	H6	7.229	7.549	8.330	7.155	---
G9	H1'	5.342	5.412	5.604	5.380	5.719
G9	H2'	2.726	2.745	2.962	2.748	2.966

G9	H2''	2.566	2.606	2.769	2.593	2.804
G9	H3'	4.935	4.916	5.069	---	5.132
G9	H4'	4.535	4.505	4.694	4.617	---
G9	H5'	---	---	---	---	---
G9	H5''	4.175	---	4.356	4.305	---
G9	P	-5.266	---	-5.037	-5.212	---
G9	H8	8.160	8.221	8.515	8.149	8.445
A10	H1'	5.976	6.059	6.140	5.992	6.241
A10	H2'	2.269	2.312	2.388	---	2.470
A10	H2''	2.329	2.361	2.431	---	2.508
A10	H3'	4.590	4.613	4.678	4.614	4.787
A10	H4'	2.088	2.125	2.207	2.129	2.394
A10	H5'	3.401	3.385	3.477	3.431	3.639
A10	H5''	3.124	3.101	3.240	3.174	3.422
A10	P	---	---	---	---	---
A10	H8	8.159	8.195	8.314	8.166	8.376
A10	H2	---	---	---	---	---
A11	H1'	6.244	6.255	6.307	6.306	6.608
A11	H2'	2.936	2.983	3.045	2.943	3.256
A11	H2''	2.843	2.885	2.934	2.891	3.191
A11	H3'	4.857	4.892	4.928	4.877	5.087
A11	H4'	4.367	---	---	---	---
A11	H5'	4.012	---	---	---	---
A11	H5''	3.858	---	---	---	---
A11	P	-5.065	---	-4.978	-5.047	---
A11	H8	7.968	8.016	8.089	8.023	8.376
A11	H2	8.093	8.117	---	8.100	8.501
G12	H1'	5.117	5.076	5.135	5.231	5.782
G12	H2'	2.736	2.759	2.782	2.795	3.193
G12	H2''	2.654	2.649	2.681	2.745	3.194
G12	H3'	4.971	4.956	4.972	5.037	5.314
G12	H4'	4.416	4.383	4.400	4.496	4.780
G12	H5'	4.291	4.282	4.313	4.346	---
G12	H5''	4.156	4.152	4.168	4.214	---
G12	P	-5.463	-5.469	-5.418	-5.435	-5.230
G12	H8	8.102	8.173	8.226	8.145	8.560
A13	H1'	6.521	6.231	6.034	6.846	---
A13	H2'	2.788	2.625	2.538	2.972	3.404
A13	H2''	3.155	2.911	2.720	3.391	3.784
A13	H3'	5.160	5.016	4.915	5.299	5.522
A13	H4'	4.601	4.447	4.355	4.757	4.971

A13	H5'	4.283	4.188	---	4.389	---
A13	H5''	4.186	4.099	4.071	4.310	4.587
A13	P	-3.679	-3.713	-3.735	-3.591	-3.305
A13	H8	8.367	8.277	8.267	8.531	9.098
A13	H2	8.124	7.679	---	8.975	---
C14	H1'	6.139	4.928	---	7.103	---
C14	H2'	2.045	1.561	---	2.396	---
C14	H2''	2.852	2.053	---	3.242	---
C14	H3'	5.002	---	---	5.238	---
C14	H4'	4.347	---	---	4.836	---
C14	H5'	---	---	---	---	---
C14	H5''	---	---	---	---	---
C14	P	-4.831	-5.045	---	-4.632	---
C14	H5	5.270	5.077	5.089	5.482	---
C14	H6	7.324	6.941	6.733	7.626	---
C15	H1'	6.417	---	---	---	---
C15	H2'	2.137	---	---	---	---
C15	H2''	3.137	---	---	---	---
C15	H3'	4.674	---	---	---	---
C15	H4'	4.527	---	---	---	---
C15	H5'	---	---	---	---	---
C15	H5''	4.269	---	---	---	---
C15	P	-3.893	---	---	-3.611	---
C15	H5	6.422	---	---	---	---
C15	H6	8.505	---	---	---	---
C16	H1'	5.505	---	---	---	---
C16	H2'	2.295	---	---	---	---
C16	H2''	1.570	---	---	---	---
C16	H3'	4.825	---	---	---	---
C16	H4'	3.713	---	---	---	---
C16	H5'	3.614	---	---	---	---
C16	H5''	3.559	---	---	---	---
C16	P	-3.870	---	---	---	---
C16	H5	6.046	---	---	---	---
C16	H6	7.595	---	---	---	---
G17	H1'	5.552	---	---	---	---
G17	H2'	2.511	---	---	---	---
G17	H2''	2.389	---	---	---	---
G17	H3'	4.316	---	---	---	---
G17	H4'	3.891	---	---	---	---
G17	H5'	---	---	---	---	---

G17	H5''	---	---	---	---	---
G17	P	-7.209	---	---	---	---
G17	H8	7.488	---	---	---	---
C18	H1'	6.038	---	---	---	---
C18	H2'	1.421	---	---	---	---
C18	H2''	2.449	---	---	---	---
C18	H3'	4.728	---	---	---	---
C18	H4'	4.258	---	---	---	---
C18	H5'	4.015	---	---	---	---
C18	H5''	3.848	---	---	---	---
C18	P	-4.290	---	---	---	---
C18	H5	4.888	---	---	---	---
C18	H6	6.818	---	---	---	---
G19	H1'	5.563	6.249	6.725	---	---
G19	H2'	2.627	3.373	---	---	---
G19	H2''	---	3.272	---	---	---
G19	H3'	4.944	5.202	5.346	---	---
G19	H4'	4.444	---	---	---	---
G19	H5'	---	---	---	---	---
G19	H5''	4.030	---	---	---	---
G19	P	-4.982	---	---	---	---
G19	H8	8.161	---	---	---	---
A20	H1'	6.031	6.460	6.559	5.652	---
A20	H2'	2.255	2.668	---	2.106	---
A20	H2''	2.343	---	---	2.107	---
A20	H3'	4.599	4.843	4.872	4.462	---
A20	H4'	2.193	2.504	---	2.065	---
A20	H5'	3.416	3.628	3.736	3.288	---
A20	H5''	3.071	3.365	3.549	2.922	---
A20	P	---	---	---	---	---
A20	H8	8.026	8.699	8.570	7.769	---
A20	H2	8.102	---	---	---	---
A21	H1'	6.424	6.503	6.714	6.298	6.714
A21	H2'	2.730	2.931	3.065	---	2.982
A21	H2''	3.009	3.136	3.304	2.917	3.252
A21	H3'	4.875	---	5.091	4.794	5.033
A21	H4'	4.401	---	---	---	---
A21	H5'	4.013	---	---	---	---
A21	H5''	3.773	---	---	---	---
A21	P	-5.110	---	---	-5.112	---
A21	H8	8.079	8.324	8.584	7.917	8.451

A21	H2	---	8.265	---	---	---
G22	H1'	5.788	5.865	6.058	5.815	6.099
G22	H2'	2.536	2.581	2.775	2.505	2.734
G22	H2''	2.840	2.882	3.062	2.833	3.045
G22	H3'	4.937	4.968	5.106	4.915	5.095
G22	H4'	4.454	4.518	4.638	4.453	4.677
G22	H5'	4.367	---	4.559	4.319	---
G22	H5''	4.197	---	4.363	4.161	---
G22	P	-5.391	---	-5.216	-5.496	-5.261
G22	H8	8.072	8.152	8.410	7.994	8.323
T23	H1'	5.901	5.898	6.104	5.999	6.181
T23	H2'	2.252	2.241	2.399	2.305	2.399
T23	H2''	2.547	2.452	2.651	2.542	2.683
T23	H3'	4.753	---	4.849	4.834	4.867
T23	H4'	4.345	---	4.471	4.364	---
T23	H5'	---	---	---	---	---
T23	H5''	4.088	---	4.204	---	---
T23	P	-5.027	---	-4.891	-5.016	-4.886
T23	Me	1.628	1.668	1.921	1.605	1.780
T23	H6	7.224	7.270	7.462	7.261	7.419
G24	H1'	5.822	5.610	5.601	5.941	5.718
G24	H2'	2.379	2.282	2.290	2.494	2.291
G24	H2''	2.665	2.538	2.502	2.769	2.536
G24	H3'	4.941	4.881	4.859	5.057	4.904
G24	H4'	4.352	4.236	---	4.481	---
G24	H5'	---	---	---	---	---
G24	H5''	---	---	---	---	---
G24	P	-4.362	---	---	-4.119	---
G24	H8	7.605	7.562	7.657	7.765	---
G25	H1'	5.966	5.839	5.676	6.024	5.554
G25	H2'	2.616	2.539	2.444	2.686	2.400
G25	H2''	2.762	2.682	2.577	2.816	2.506
G25	H3'	5.004	4.922	4.817	5.069	4.814
G25	H4'	4.438	4.296	4.114	4.524	4.141
G25	H5'	---	---	---	---	---
G25	H5''	---	---	---	---	---
G25	P	-4.666	-4.712	-4.816	-4.489	-4.760
G25	H8	7.815	7.736	7.668	7.910	7.615
C26	H1'	5.986	5.930	5.821	5.987	5.617
C26	H2'	2.057	2.005	1.930	2.059	1.815
C26	H2''	2.460	2.424	2.339	2.457	2.205

C26	H3'	4.838	4.793	4.697	4.847	4.599
C26	H4'	4.211	4.149	---	---	3.840
C26	H5'	---	---	---	---	---
C26	H5''	4.170	---	---	---	---
C26	P	-4.630	-4.710	---	-4.582	-4.863
C26	H5	5.295	5.258	5.192	5.361	5.102
C26	H6	7.363	7.298	7.220	7.388	7.111
C27	H1'	5.662	5.639	5.597	5.651	5.466
C27	H2'	2.006	1.994	1.940	2.002	1.843
C27	H2''	2.356	2.345	2.308	2.347	2.204
C27	H3'	4.843	4.836	4.782	4.835	4.681
C27	H4'	4.123	4.117	4.048	4.108	3.903
C27	H5'	---	---	---	---	---
C27	H5''	4.095	---	---	---	---
C27	P	-4.566	-4.592	-4.679	-4.558	-4.797
C27	H5	5.666	5.636	5.594	5.684	5.502
C27	H6	7.482	7.453	7.404	7.483	7.295
G28	H1'	6.214	6.205	6.201	6.205	6.105
G28	H2'	2.661	2.657	2.645	2.652	2.564
G28	H2''	2.407	2.399	2.391	2.397	2.318
G28	H3'	4.715	4.712	---	4.705	4.621
G28	H4'	4.215	4.216	---	4.203	4.101
G28	H5'	---	---	---	---	---
G28	H5''	4.104	4.107	---	---	---
G28	P	-4.208	-4.216	---	-4.221	-4.348
G28	H8	7.986	7.975	7.953	7.981	7.870

Table S3. Structure determination statistics for LnA_28.

PDB ID	7QB3
Structures calculated	100
Structures deposited	20
**No. of restraints**	
Total	879
NOE distance restraints	221
Intra-residue	101
Sequential residues	107
Long range	13
Hydrogen bond	66*
Dihedral restraints	109
PCS restraints	483
**Structural refinement statistics**	
Violations of experimental restraints	
NOE distance restraints	
Number of violations > 0.5 Å	0
Mean NOE violation (Å)	0.06 (0.01)
Dihedral restraints	
Number of violations > 30°	0
Mean dihedral violation (°)	3.2 (0.7)
PCS restraints	
Q_{Eu3+}	0.074 (0.006)
Q_{Yb3+}	0.068 (0.005)
Q_{Ce3+}	0.135 (0.007)
Q_{Tm3+}	0.067 (0.005)
Mean deviations from idealized geometry	
Bonds (Å)	0.01 (0.00)
Angles (deg)	1.63 (0.02)
Pairwise RMSD (Å)	0.70 (0.11)

*33 hydrogen bonds imposed, with two restraints per bond

Table S4 Occupancies of hydrogen bonds between water molecules directly coordinating a Ln^{3+} ion and electronegative atoms within LnA_28 observed in the performed MD simulations. See Figure 7 for the geometric details of each hydrogen bonding interaction.

		Metal ion present in the simulation				
		La^{3+}	Ce^{3+}	Eu^{3+}	Tm^{3+}	Lu^{3+}
Hydrogen bond acceptor	G19 - O6	77.5%	64.7%	36.7%	67.5%	61.7%
	G19 - N7	51.3%	53.2%	61.1%	24.7%	16.0%
	G6 - N3	83.5%	86.4%	89.5%	88.6%	88.5%
	T7 - O4’	64.3%	67.8%	67.4%	63.7%	60.9%
	G17 - OP2	98.1%	96.1%	61.9%	96.7%	96.4%
	C18 - OP1	47.2%	49.1%	33.5%	79.0%	78.8%
Metal coordination number		9	9	7 (29%) or 8 (49 %) or 9 (19 %)	8 (89%) or 9 (11%)	8
